# Gut microbiota-derived indole 3-propionic acid protects against radiation toxicity via retaining acyl-CoA-binding protein

**DOI:** 10.1186/s40168-020-00845-6

**Published:** 2020-05-20

**Authors:** Hui-wen Xiao, Ming Cui, Yuan Li, Jia-li Dong, Shu-qin Zhang, Chang-chun Zhu, Mian Jiang, Tong Zhu, Bin Wang, Hai-Chao Wang, Sai-jun Fan

**Affiliations:** 1 Tianjin Key Laboratory of Radiation Medicine and Molecular Nuclear Medicine, Institute of Radiation Medicine, Chinese Academy of Medical Sciences and Peking Union Medical College, 238 Baidi Road, Tianjin, 300192 China; 2 Laboratory of Emergency Medicine, Feinstein Institute for Medical Research, Manhasset, NY USA

**Keywords:** Radiotherapy, Acute radiation syndrome, Gastrointestinal tract toxicity, Hematopoietic toxicity, Gut microbiota, Gut microbiota metabolite, Indole 3-propionic acid, ACBP

## Abstract

**Background:**

We have proved fecal microbiota transplantation (FMT) is an efficacious remedy to mitigate acute radiation syndrome (ARS); however, the mechanisms remain incompletely characterized. Here, we aimed to tease apart the gut microbiota-produced metabolites, underpin the therapeutic effects of FMT to radiation injuries, and elucidate the underlying molecular mechanisms.

**Results:**

FMT elevated the level of microbial-derived indole 3-propionic acid (IPA) in fecal pellets from irradiated mice. IPA replenishment via oral route attenuated hematopoietic system and gastrointestinal (GI) tract injuries intertwined with radiation exposure without precipitating tumor growth in male and female mice. Specifically, IPA-treated mice represented a lower system inflammatory level, recuperative hematogenic organs, catabatic myelosuppression, improved GI function, and epithelial integrity following irradiation. 16S rRNA gene sequencing and subsequent analyses showed that irradiated mice harbored a disordered enteric bacterial pattern, which was preserved after IPA administration. Notably, iTRAQ analysis presented that IPA replenishment retained radiation-reprogrammed protein expression profile in the small intestine. Importantly, shRNA interference and hydrodynamic-based gene delivery assays further validated that pregnane X receptor (PXR)/acyl-CoA-binding protein (ACBP) signaling played pivotal roles in IPA-favored radioprotection in vitro and in vivo.

**Conclusions:**

These evidences highlight that IPA is a key intestinal microbiota metabolite corroborating the therapeutic effects of FMT to radiation toxicity. Owing to the potential pitfalls of FMT, IPA might be employed as a safe and effective succedaneum to fight against accidental or iatrogenic ionizing ARS in clinical settings. Our findings also provide a novel insight into microbiome-based remedies toward radioactive diseases.

Video abstract.

## Background

Cancer is a leading cause of death throughout the world. Despite considerable advances in understanding the molecular basis of abdominal and pelvic neoplasms, abdominal and pelvic cancers, such as colorectal cancer, prostate cancer, and cervical cancer, remain the most common form of cancer leading tumor-related mortality globally [1–3]. As a common feasible therapy approach, about 50–60% of cancer patients receive radiotherapy during their treatment courses [4]. Radiotherapy represents the most effective therapeutic regimen for patients with cancer and improves their survival. However, the final outcome of this single treatment modality is still uncertain, with a high risk of recurrence among patients with unfavorable side effects [5]. After radiation exposure, a complex array of clinical complications are accompanied, such as bone marrow toxicity (hematopoietic syndrome) and gastrointestinal toxicity (GI syndrome), which are collectively known as acute radiation syndrome (ARS) [6]. Even for healthy populations, unwanted radiological or nuclear exposure remains a serious public health risk [7]. Unfortunately, effective countermeasure agents to attenuate ARS in exposed individuals remain an unmet medical need.

The ecosystem of mammalian GI tract emerges as home to trillions of microbes, including bacteria, archaea, viruses, and fungi, which collectively termed as gut microbiota [8].The major function of gut microbiota is to aid in the harvest of nutrients and energy from our varied diets [9]. Furthermore, it influences a range of metabolic, developmental, and physiological processes affecting host health through stimulating the development of hosts’ immune system [10–12], protecting against pathogen invasion [13], and regulating brain development and behavior [14]. The mammalian GI tract is an essential mutualism exists within the intestinal mucosa [15]. Accordingly, the gut microbiota exerts its effects by producing bioactive compounds [16]. These microbiota-derived metabolites signal to distant organs in the body, which enables the enteric bacteria to connect to the immune and hormone system [17], brain (the gut-brain axis) [18], and host metabolism, as well as other functions [19]. Indole 3-propionic acid (IPA) is an enteric microbiome-derived deamination product of tryptophan and performs intracellular signaling activity [20]. However, whether gut microbiota metabolites, such as IPA, play a part in alleviating ARS and the underlying molecular mechanism are elusive.

Acyl-CoA-binding protein (ACBP)/diazepam-binding inhibitor (DBI) (hereafter named ACBP) is a 10-kDa intracellular protein expressing in all eukaryotic species and mammalian tissues investigated [21]. ACBP is expressed at relatively high levels in the epidermis, particularly in the suprabasal layers, which are highly active in lipid synthesis [22]. In vitro studies indicate that ACBP induces steroidogenesis in isolated adrenal mitochondria, inhibits glucose induced insulin secretion from the pancreas, induces medium-chain acyl-CoA ester synthesis, and affects cell growth [23, 24]. In addition, whole body ACBP knock-out mice have impaired anxiolytic responses to diazepam [25]. Pregnane X receptor (PXR) is a ligand-activated nuclear receptor sensing and responding to a spectrum of chemical or nutritional stimuli including circulating IPA [26]. However, whether IPA modulates the expression of ACBP through PXR required to be documented.

In this study, we sought to investigate whether IPA, a bacterial-mediated production from tryptophan, ameliorates ARS using mouse models. Our observations demonstrated that oral gavage of IPA protected against bone marrow and GI toxicity intertwined with radiation exposure. Mechanistically, IPA administration preserved the intestinal bacterial composition structure and retained the protein profile disturbed by irradiation. Importantly, PXR/ACBP axis was essential to IPA exerted radioprotective function. Collectively, our findings provide new insights into the function and the underlying protective mechanism of microbiota metabolites in the context of ARS in a preclinical setting.

## Results

### IPA replenishment protects against radiation-induced mortality in vitro and in vivo

**Fig. 1:**
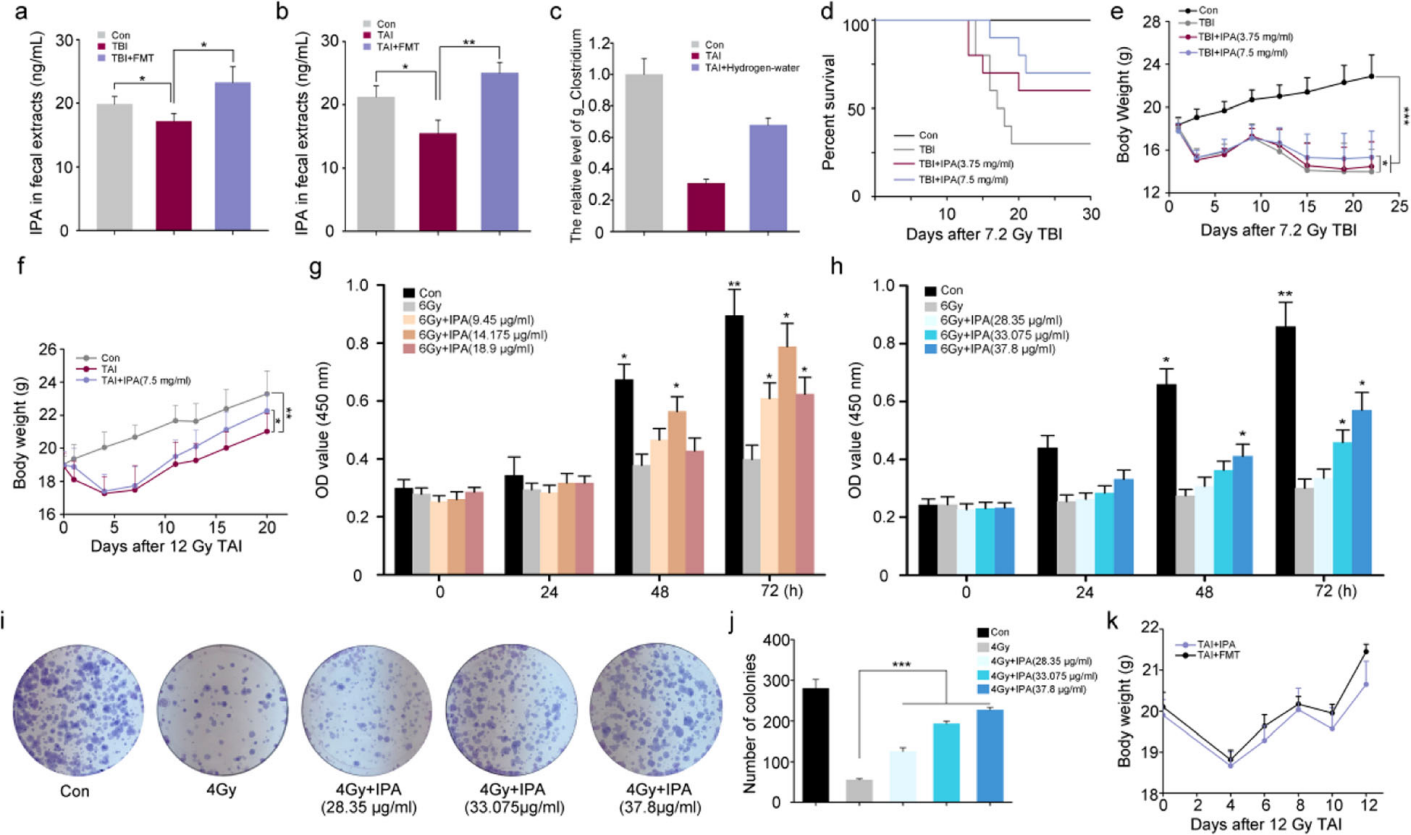
IPA replenishment protects against radiation-induced mortality in vitro and in vivo. **a**, **b** The concentrations of IPA in fecal pellets from each cohort was measured at the end of receiving 10 days of FMT. The IPA levels are not significantly different between control vs TAI + FMT or TBI + FMT. Significant differences between each two cohorts are indicated: *P < 0.05 and **P < 0.01; Student’s *t* test. **c** The relative abundance of g_*Clostridium* was compared among control, TAI, and TAI + hydrogen-water groups through 16S rRNA sequencing analysis. **d** Kaplan-Meier analysis of male mice treated with the indicated irradiation and with IPA or saline. *n* = 24 per group. *P < 0.05 by log-rank test between 7.5 mg/ml IPA and TBI groups. **e**, **f** Body weights were compared among male mice after 7.2 Gy TBI or 12 Gy TAI, *n* = 24 per group; Significant differences between each two cohorts are indicated: *P < 0.05, **P < 0.01, and ***P < 0.005; Student’s *t* test. **g**–**i** The effects of concentration gradient IPA on the proliferation of MODE-K cells (**g**) and HIEC-6 cells (**h**–**j**) were assessed by CCK-8 assays and cloning formation assays, respectively. Significant differences between each two cohorts are indicated: *P < 0.05, **P < 0.01, and ***P < 0.005; Student’s *t* test. **k** Body weights were compared between FMT group and IPA group after 12 Gy TAI, *n* = 24 per group

Previously, we reported fecal microbiota transplantation (FMT) could protect against ARS [27]. On the basis of the study, we tested intestinal microbial metabolites from mouse fecal extracts. Notably, both total body and abdominal irradiation exposure lessened the content of IPA, while FMT erased the alterations (Fig. 1a, b). In addition, the untargeted metabolomics KEGG analysis also enriched into the pathway of indole alkaloid biosynthesis (Additional file 1: Figure S1A, B). In the survey of IPA production by representative members of the intestinal microbiota, only *Clostridium* was found to produce IPA [28]. Thus, we compared the frequency of *Clostridium* in mouse fecal extracts based on our previous study [29]. 16S rRNA sequencing analysis showed that total abdominal irradiation exposure lessened the level of *Clostridium*, while GI toxicity rehabilitation was enmeshed with elimination of the shifts (Fig. 1c). Initially, we identified whether IPA replenishment could protect against death from exposure to irradiation. After exposure to 7.2 Gy total body irradiation (TBI), the male animal survival rate was decreased by 60% in the control vehicle group, but it was decreased by 50% or 30% in irradiation male animals receiving 3.75 or 7.5 mg/ml concentration of IPA (via oral route, Fig. 1d). Body weight loss is considered as a treatment toxicity, and IPA administration also increased the body weight of irradiated mice after TBI or total abdominal irradiation (TAI) in a dose-dependent fashion (Fig. 1e, f, and 7.5 mg/ml of IPA was used as the optimal concentration in the following study), indicating that IPA replenishment protects against radiation-induced mortality and weight loss. In vitro, IPA facilitated the proliferation of irradiated MODE-K and HIEC-6 cells in a dose-dependent manner, as demonstrated by cell counting kit-8 (CCK-8) assays (Fig. 1g, h and Additional file 1: Figure S1C, D) and cloning formation (Fig. 1i, j). Of note, the body weight of TAI-exposed mice treated with FMT or IPA showed no significant difference, indicating that IPA replenishment might mimic FMT to mitigate radiation injuries (Fig. 1k). Thus, we conclude that IPA administration protects against radiation-induced mortality in vitro and in vivo.

### Oral gavage of IPA ameliorates TBI-associated hematopoietic system injury

**Fig. 2:**
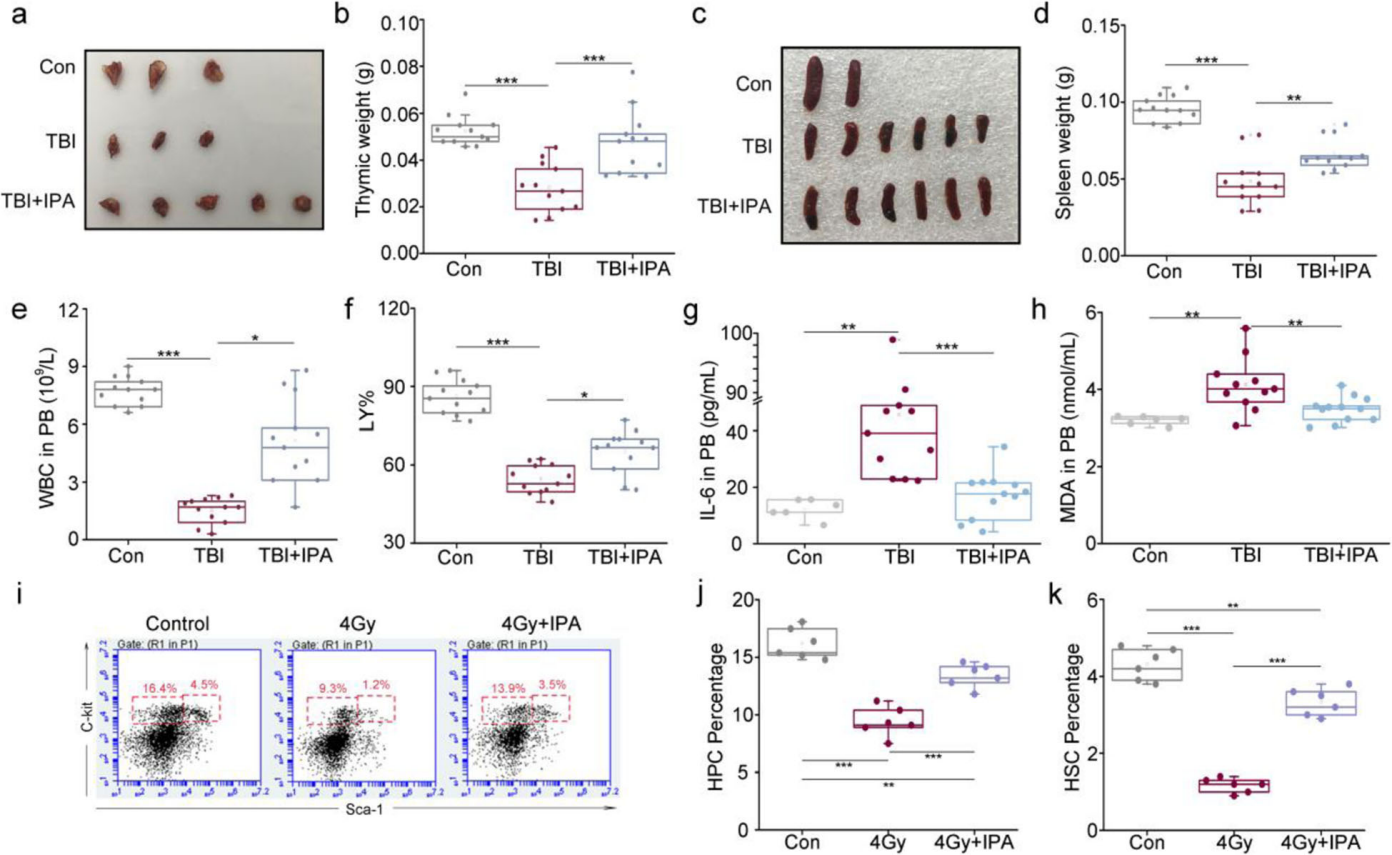
Oral gavage of IPA ameliorates TBI-associated hematopoietic system injury. **a**, **b** Photographs (**a**) and weight (**b**) of dissected thymuses from mice in the three groups, the thymuses were obtained at day 15 after 4 Gy TBI. Mean ± SEM. Significant differences between each two cohorts are indicated: ***P < 0.005; Student’s *t* test, *n* = 12 per group. **c**, **d** Photographs (**c**) and weight (**d**) of the dissected spleens from mice in the three groups, the spleens were obtained at day 15 after 4 Gy TBI. Significant differences between each two cohorts are indicated: **P < 0.01 and ***P < 0.005; Student’s *t* test, *n* = 12 per group. **e**, **f** White blood cell (WBC) counts (**e**) and percentage of lymphocytes (LY%) (**f**) in PB were measured at day 15 after 4 Gy TBI. The data were presented as means ± SEM (*n* = 12 per group). Significant differences between each two cohorts are indicated: *P < 0.05 and ***P < 0.005; Student’s *t* test. **g**, **h** The content of IL-6 (**g**) and MDA (**h**) in PB were examined. Mean ± SEM. Significant differences between each two cohorts are indicated: **P < 0.01 and ***P < 0.005; Student’s *t* test, *n* = 6 for control group; *n* = 11 for TBI group; *n* = 12 for TBI + IPA group. **i**–**k** Representative FACS plots of HSCs, HPCs. The percentage of hematopoietic progenitor cells (HPCs) and HSC cells in lineage-negative cells were analyzed at day 15 after 4 Gy TBI. The data were presented as means ± SEM (*n* = 6 per group). Significant differences between each two cohorts are indicated: **P < 0.01 and ***P < 0.005; Student’s *t* test

Given the hematopoietic system is especially sensitive to total body irradiation representing as atrophic hematogenic organs and a massive loss of hematopoietic stem cells in mouse models, we addressed the protective effects of IPA on hematopoietic system in male mice. Four gray of TBI reduced the volumes and weight of thymus and spleen, which restored by IPA treatment (Fig. 2a–d). Peripheral blood (PB) analysis revealed that the irradiated mice exhibited a significant decrease in WBC counts, RBC counts, HGB, percentage of neutrophil granulocytes (NE%), and lymphocytes (LY%); nevertheless, oral gavage of IPA attenuated the decrease of those in peripheral blood (Fig. 2e, f and Additional file 1: Figure S2A-C). Inflammatory markers (*IL-6* and *TNFɑ*) as well as oxidative stress marker (*MDA*) were significantly elevated in PB from irradiated animals, which were reduced following IPA treatment (Fig. 2g, h and Additional file 1: Figure S2D). Hematopoietic stem and progenitor cell (HSPCs) exhaustion has been proposed to be primarily responsible for myelosuppression induced by TBI [30]. To determine whether IPA ameliorates myelosuppression by inhibiting HSPC exhaustion, we analyzed the HSC cells (Lin^−^Sca-l^+^c-kit^+^) and HPCs (Lin^−^Sca-l^−^c-kit^+^) in BM cells 15 days after 4 Gy TBI. As shown in Fig. 2i–k, IPA treatment increased percentages of HSC cells and HPCs compared to saline-treated mice which suggested that IPA significantly increased the recovery of BM HPCs/HSC cells after TBI. Together, our observations demonstrated that IPA replenishment could ameliorate TBI-accompanied hematopoietic system injury.

### IPA administration improves GI tract toxicity after total abdominal irradiation

**Fig. 3:**
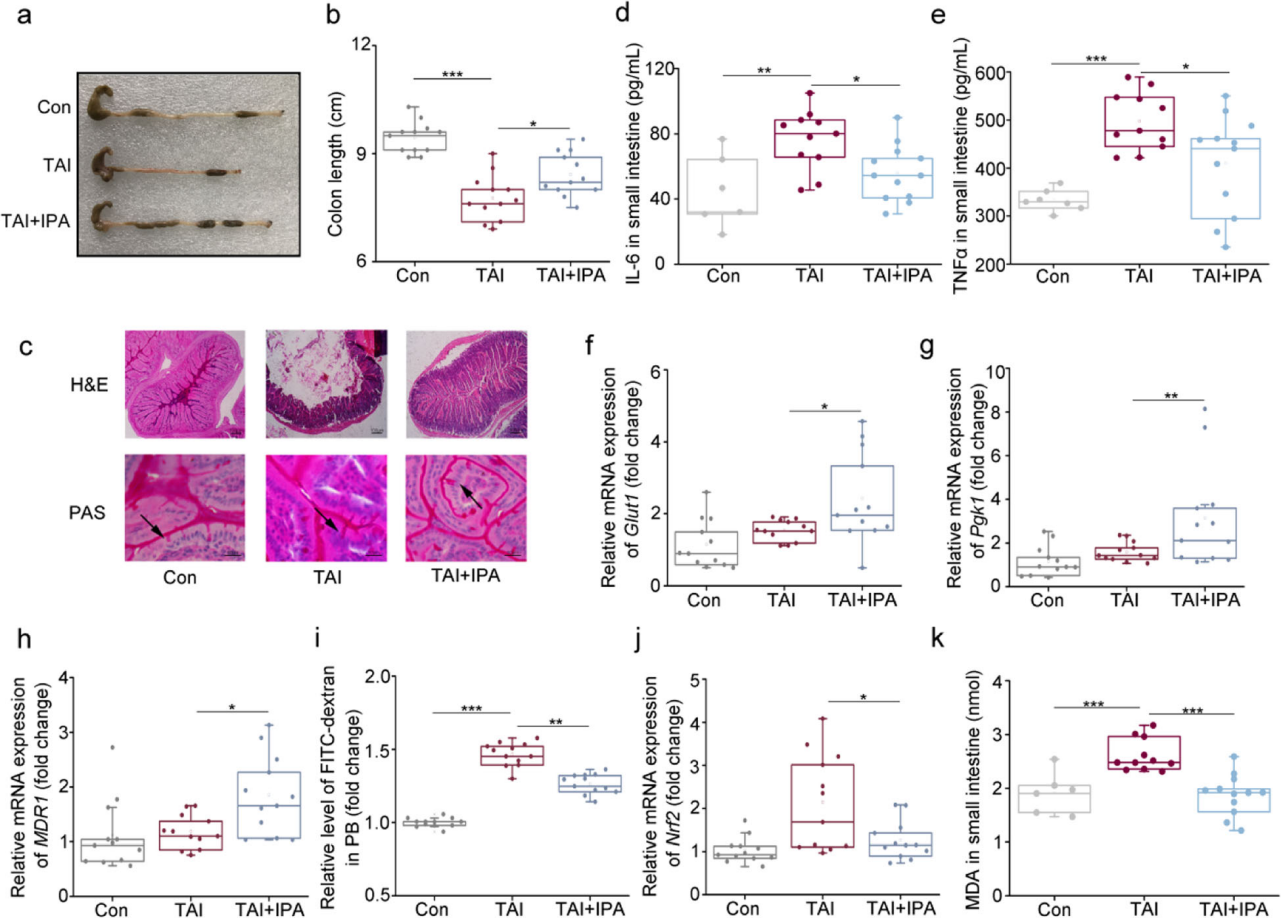
IPA administration improves GI tract toxicity after total abdominal irradiation. **a**, **b** Photographs (**a**) and length (**b**) of dissected colon from mice in the three groups, the colon tissues were obtained at day 21 after 12 Gy TAI. Mean ± SEM. Significant differences between each two cohorts are indicated: *P < 0.05 and ***P < 0.005; Student’s *t* test, *n* = 12 per group. **c** The morphology of the small intestine was shown by H&E (×100 magnification; scale bar: 100 μm) and PAS (×1000 magnification; scale bar: 50 μm) staining. The small intestine tissues were obtained at day 21 after 12 Gy TAI. The arrows point to the goblet cells. **d**, **e** The content of *IL-6* (**d**) and *TNFɑ* (**e**) in the small intestine tissues were examined by ELISA. Mean ± SEM. Significant differences between each two cohorts are indicated: *P < 0.05, **P < 0.01, and ***P < 0.005; Student’s *t* test, *n* = 6 for control group, *n* = 11 for TAI group, *n* = 12 for TAI + IPA group. **f**–**h** The expression levels of *Glut1* (**f**), *Pgk1* (**g**), and *MDR1* (**h**) were examined in the small intestine tissues by qRT-PCR. The small intestine tissues were obtained at day 21 after 12 Gy TAI. Mean ± SEM. Significant differences between each two cohorts are indicated: *P < 0.05 and **P < 0.01; Student’s *t* test, *n* = 12 per group. **i** The FITC-dextran in PB was assessed at day 21 after 12 Gy TAI. Mean ± SEM. Significant differences between each two cohorts are indicated: **P < 0.01 and ***P < 0.005; Student’s *t* test, *n* = 12 per group. **j** The expression levels of *Nrf2* was assessed in the small intestine tissue by qRT-PCR. The small intestine tissues were obtained at day 21 after 12 Gy TAI. Mean ± SEM. Significant differences between each two cohorts are indicated: *P < 0.05; Student’s *t* test, *n* = 12 per group. **k** The content of MDA in the small intestine tissues were examined. Mean ± SEM. Significant differences between each two cohorts are indicated: ***P < 0.005; Student’s *t* test, *n* = 6 for control group, *n* = 11 for TAI group, *n* = 12 for TAI + IPA group

The small intestine is also the major sites of injury during radiation therapy, representing as enteritis, loss of GI tract structure, and barrier function in mouse models. To examine the protective effects of IPA on radiation-induced GI injury, we compared the colon length and histologically small intestines from irradiated male mice with or without IPA administration. As expected, IPA replenishment reversed shortening of colon, losing of intestinal villi, and decreasing of goblet cells, which were observed in radiation group (Fig. 3a–c). Radiation-elevated inflammatory cytokines, such as *IL-6* and *TNFɑ*, were reduced in TAI-exposed mice following IPA administration, suggesting that IPA ameliorated radiation-induced enteric inflammation (Fig. 3d, e and Additional file 1: Figure S3A, B). We further validated that the expression of *Glut1* (*Slc2a1*), *Pgk1*, and multidrug resistance protein 1 (*MDR1*), which all participated in epithelial integrity maintaining after toxic stimuli [31], reached about twofold higher levels in the small intestine tissues from irradiated mice with IPA treatment (Fig. 3f–h). In addition, IPA administration decreased the radiation-heightened FITC-dextran level in PB (Fig. 3i), indicating that IPA improves GI tract barrier function and epithelial integrity in irradiated animals. Massive production of reactive oxygen species (ROS) is a shared feature of radiation stimuli, as our results showed that oral gavage of IPA blunted the radiation-heightened *Nrf2* and *MDA* levels in the small intestine tissues (Fig. 3j, k). Together, IPA replenishment ameliorates radiation-caused GI toxicity by enhancing intestinal barrier function and epithelial integrity, hindering radiation-induced inflammation, and reducing ROS levels.

### IPA protects against radiation toxicity in mouse models without accelerating tumor growth

**Fig. 4:**
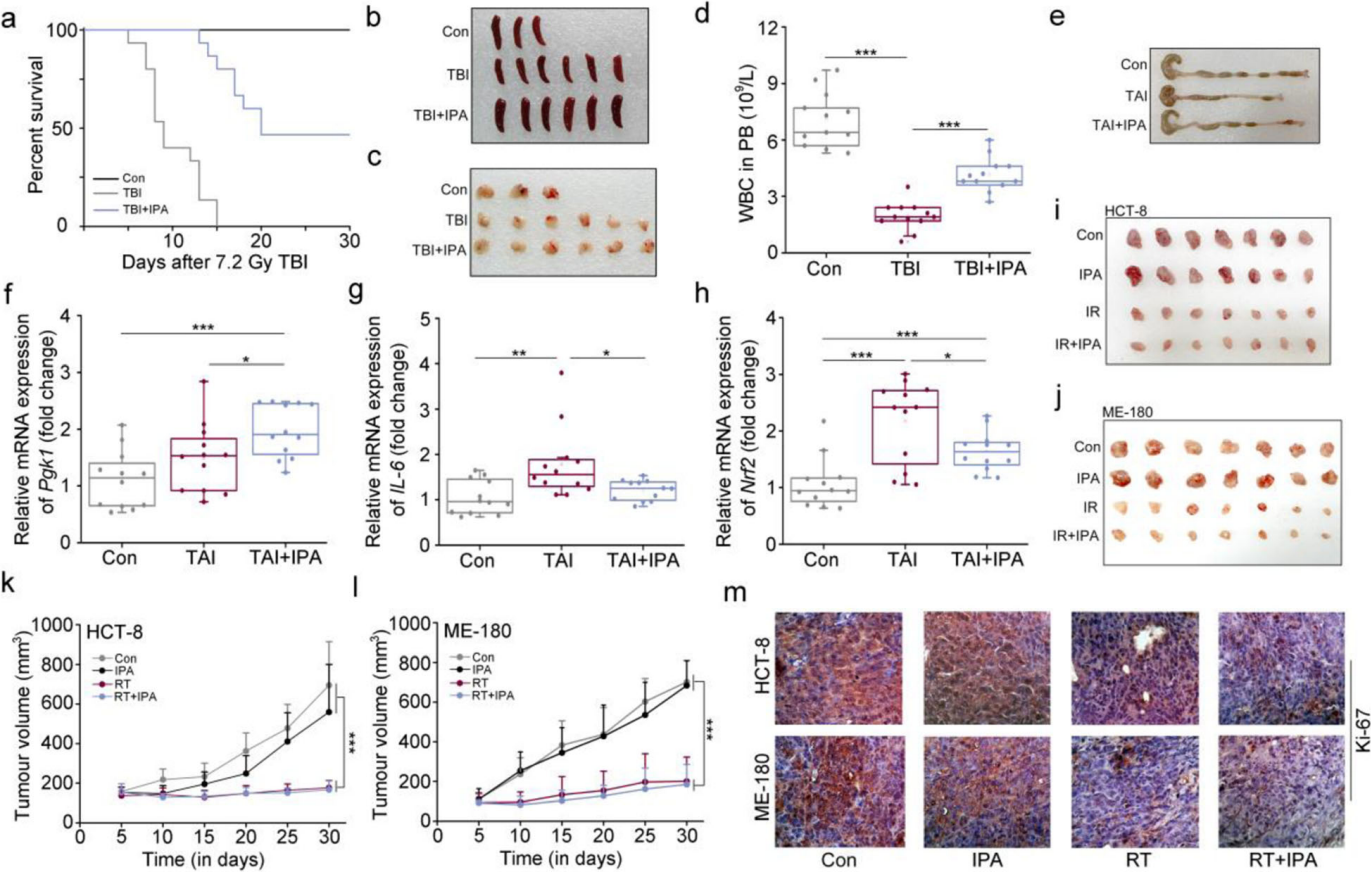
IPA protects against radiation toxicity in mouse models without accelerating tumor growth. **a** Kaplan-Meier analysis of female mice in the three groups after 7.2 Gy TBI, *n* = 30 per group. *P < 0.05 by log-rank test between IPA and TBI groups. **b**, **c** Photographs of dissected the thymuses and spleens from female mice in the three groups, and the thymuses and spleens were obtained at day 15 after 4 Gy TBI. *n* = 12 per group. **d** White blood cell (WBC) counts in PB from female mice were measured at 15 days after 4 Gy TBI. The data were presented as means ± SEM (*n* = 12 per group). Significant differences between each two cohorts are indicated: ***P < 0.005; Student’s *t* test. **e** Photographs of dissected colon from female mice in the three groups, and the colon tissues were obtained at day 21 after 12 Gy TAI. *n* = 12 per group. **f–h** The expression levels of *Pgk1* (**f**), *IL-6* (**g**), and *Nrf2* (**h**) were examined in the small intestine tissues by qRT-PCR. The small intestine tissues were obtained at day 21 after 12 Gy TAI. Mean ± SEM. Significant differences between each two cohorts are indicated: *P < 0.05, **P < 0.01, and ***P < 0.005; Student’s *t* test, *n* = 12 per group. **i**, **j** The growth images of HCT-8 and ME-180 cells in nude mice administrated with IPA and local radiation. **k**, **l** The growth curve of HCT-8 and ME-180 cells in nude mice administrated with IPA and local radiation. Data are expressed as mean ± SEM from 7 mice. Statistically significant differences between each two cohorts are indicated: ***P < 0.001; Student’s *t* test. **m** The expressions of Ki-67 were examined by immunohistochemistry staining in HCT-8 and ME-180 tumor tissues from nude mice

To further identify the radioprotection of IPA, female mice were treated with IPA following TBI or TAI challenge via oral route. In parallel with their male counterparts, IPA replenishment mitigated radiation-induced hematopoietic and GI syndromes in female mice. In detail, IPA administration elevated survival rate and body weight (Fig. 4a and Additional file 1: Figure S4A), restored the atrophic thymus and spleen (Fig. 4b, c and Additional file 1: Figure S4B, C), and heightened WBC counts and percentage of lymphocytes in PB after radiation exposure (Fig. 4d and Additional file 1: Figure S4D). For GI toxicity, IPA treatment prolonged colon length (Fig. 4e and Additional file 1: Figure S4E), recovered intestinal villi and goblet cells (Additional file 1: Figure S4F), facilitated small intestinal integrity (Fig. 4f and Additional file 1: Figure S4G), and hindered enteritis (Fig. 4g and Additional file 1: Figure S4H) and ROS level (Fig. 4h). Together, our observations demonstrate that gut microbiota-derived IPA protects both sexes from ARS.

To investigate whether IPA can be employed to improve prognosis of radiotherapy in clinical application, we further examined whether IPA precipitated cancer cell proliferation in vivo. Male (or female) BALB/c athymic nude mice were injected with HCT-8 (or ME-180) cells subcutaneously and treated with IPA with or without local radiation. Intriguingly, IPA replenishment did not increase the volume and weight of the tumors in animals receiving exogenous cancer cells (Fig. 4i–l and Additional file 1: Figure S4I, J). Immunohistochemical staining further validated that IPA unaltered the expression of Ki-67, a marker of proliferation, in tumor tissues with or without radiation challenge (Fig. 4m), indicating that IPA treatment do not accelerate tumor growth.

### IPA treatment changes irradiation-shaped intestinal bacterial structure

Next, we aimed to elucidate the underlying mechanism of radioprotection by IPA. Given gut microbiota configurations relate to ARS progression, we addressed the effects of IPA on the alterations of intestinal bacterial structure in TAI-exposed male mice. At day 6 after radiation exposure, the observed species number of enteric bacteria among control, TAI-exposed, and TAI-exposed with IPA replenishment mice was unchanged (Additional file 1: Figure S5A, B). However, an unweighted principle coordinate analysis (PCoA), principal component analysis (PCA), and non-metric multidimensional scaling (NMDS) analysis were conducted to visualize differences in bacterial taxa composition among the three groups (Additional file 1: Figure S5C-E). Statistically, unweighted unifrac analysis but not weighted revealed that TAI drove a marked difference in gut microbiota composition, whereas IPA administration narrowed the alterations (Additional file 1: Figure S5F, G), suggesting that IPA might preserve TAI-shifted bacterial composition to perform radioprotection. In details, TAI treatment caused a lower relative abundance of *Lactobacillus* at the genus level (Additional file 1: Figure S5H, I) and a higher relative abundance of *g*_*Bacteroides*_*acidifaciens* and *Ruminoccoccus*_*gauvreauii* at the species level (Additional file 1: Figure S5J, K), whereas IPA administration reversed these changes. Linear discriminant analysis effect size (LEfSe) assays exhibited the increases in *Blautia* (genus), *Proteobacteria* (phylum), and *Parabacteroides* (genus) and decreases in *Bacteroides* (genus), *Enterobacterides* (order), and *Escherichia coli* (species) which are the main altered microbes following IPA administration in irradiated mice (Additional file 1: Figure S5L, M).

**Fig. 5:**
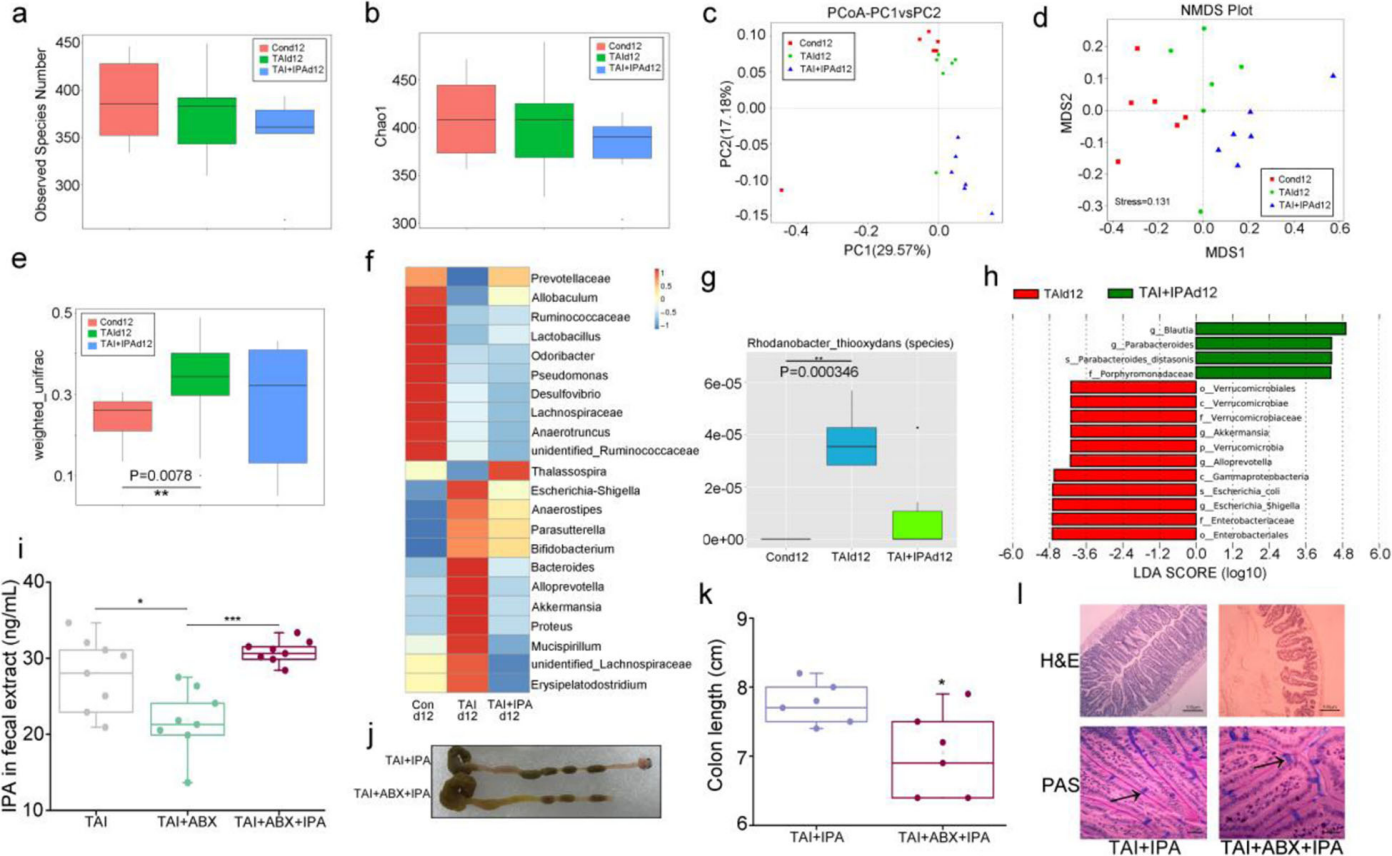
IPA preserves irradiation-shifted enteric bacterial composition at day 12 after TAI. **a**, **b** The observed species number and Chao1 diversity index of intestinal bacteria was examined by 16S rRNA high-throughput sequencing after 12 days of TAI exposure. Significant differences are indicated: Wilcoxon rank sum test. *n* = 6 per group. **c**, **d** PCoA and NMDS were used to measure the shift in intestinal bacterial composition profile after irradiation at day 12. **e** The *β* diversity of intestinal bacteria was compared by the weighted unifrac analysis. Significant differences are indicated: Wilcoxon rank sum test. *n* = 6 per group. **f** The alteration of intestinal bacterial patterns at the genus level was assessed by 16S rRNA sequencing, *n* = 6 per group. The heat map is color-based on row *Z*-scores. The mice with the highest and lowest bacterial level are in red and blue, respectively. **g** The abundances of most varied strain bacteria was assessed using 16S high-throughput sequencing after irradiation at day 12. Statistically significant differences are indicated: Wilcoxon rank sum test, *n* = 6 per group. **h** Linear discriminant analysis (LDA) effect size (LEfSe) results represented significantly different in abundance of gut bacteria between TAI and IPA groups and indicated the effect size of each differentially abundant bacterial taxon in the small intestine after irradiation at day 12, *n* = 6 per group. Significant differences are indicated: Wilcoxon rank sum test. **i** The content of IPA in fecal was examined by ELISA. Mean ± SEM. Significant differences between each two cohorts are indicated: *P < 0.05 and ***P < 0.005; Student’s *t* test, *n* = 8 per group. **j**, **k** Photographs (**j**) and length (**k**) of dissected colon from IPA gavage mice with or without antibiotics (ABX) treatment, the colon tissues were obtained at day 21 after 12 Gy TAI. Mean ± SEM. Significant differences are indicated: Student’s *t* test, *n* = 6 per group. **l** The morphology of the small intestine was shown by H&E (× 100 magnification; scale bar: 100 μm) and PAS (× 1000 magnification; scale bar: 50 μm) staining. The small intestine tissues were obtained at day 21 after 12 Gy TAI. The arrows point to the goblet cells

At day 12 after TAI exposure, the observed species numbers of intestinal bacteria showed no difference among the three cohorts (Fig. 5a, b). Although unweighted unifrac analysis described no changes of the gut microbiota composition statistically (Additional file 1: Figure S6A), PCA, PCoA, and NMDS plot indicated an obvious separation after IPA administration in irradiated mice (Fig. 5c, d and Additional file 1: Figure S6B). Weighted unifrac analysis revealed that TAI drove a marked difference in gut microbiota composition, whereas IPA administration reduced the alterations (Fig. 5e), suggesting that IPA might preserve TAI-shaped bacterial composition to perform radioprotection. In detail, however, TAI exposure kept the relative abundance of *Bacteroides* (at the genus level, Fig. 5f and Additional file 1: Figure S6C) and *Rhodanobacter_thiooxydans* (at the species level, Fig. 5g) at a higher level; IPA replenishment retained that of *Bacteroides* and *Rhodanobacter_thiooxydans* (Fig. 5f, g and Additional file 1: Figure S6C). LEfSe assays further indicated that the *Enterobacteriales* became more abundant after radiation exposure compared to IPA group, in which *Blautia* and *Porphyromonadaceae* were more abundant (Fig. 5h and Additional file 1: Figure S6D). Collectively, these results indicate that oral gavage of IPA changes irradiation-disordered intestinal bacterial structure.

Since IPA impacted intestinal bacterial structure, we are curious about whether IPA mitigates radiation-induced GI toxicity depending on enteric microflora. Thus, the male mice were treated with antibiotics (ABX) to clear gut microbes. As expected, ABX treatment lessened the content of IPA in fecal pellets, while IPA gavage abrogated the reduction (Fig. 5i). Importantly, IPA replenishment failed to attenuate radiation-caused GI toxicity in ABX-challenged mice, manifested as shortening of colon (Fig. 5j, k), losing of intestinal villi (Fig. 5l, first line) and decreasing of goblet cells (Fig. 5l, second line). Furthermore, ABX treatment eradicated IPA replenishment rescued weight loss, enteric inflammation, and ROS levels (Additional file 1: Figure S7), indicating that IPA protects against radiation-induced GI injuries partly based on gut microbes.

### IPA replenishment reprograms small intestinal protein expression profile following TAI challenge

**Fig. 6:**
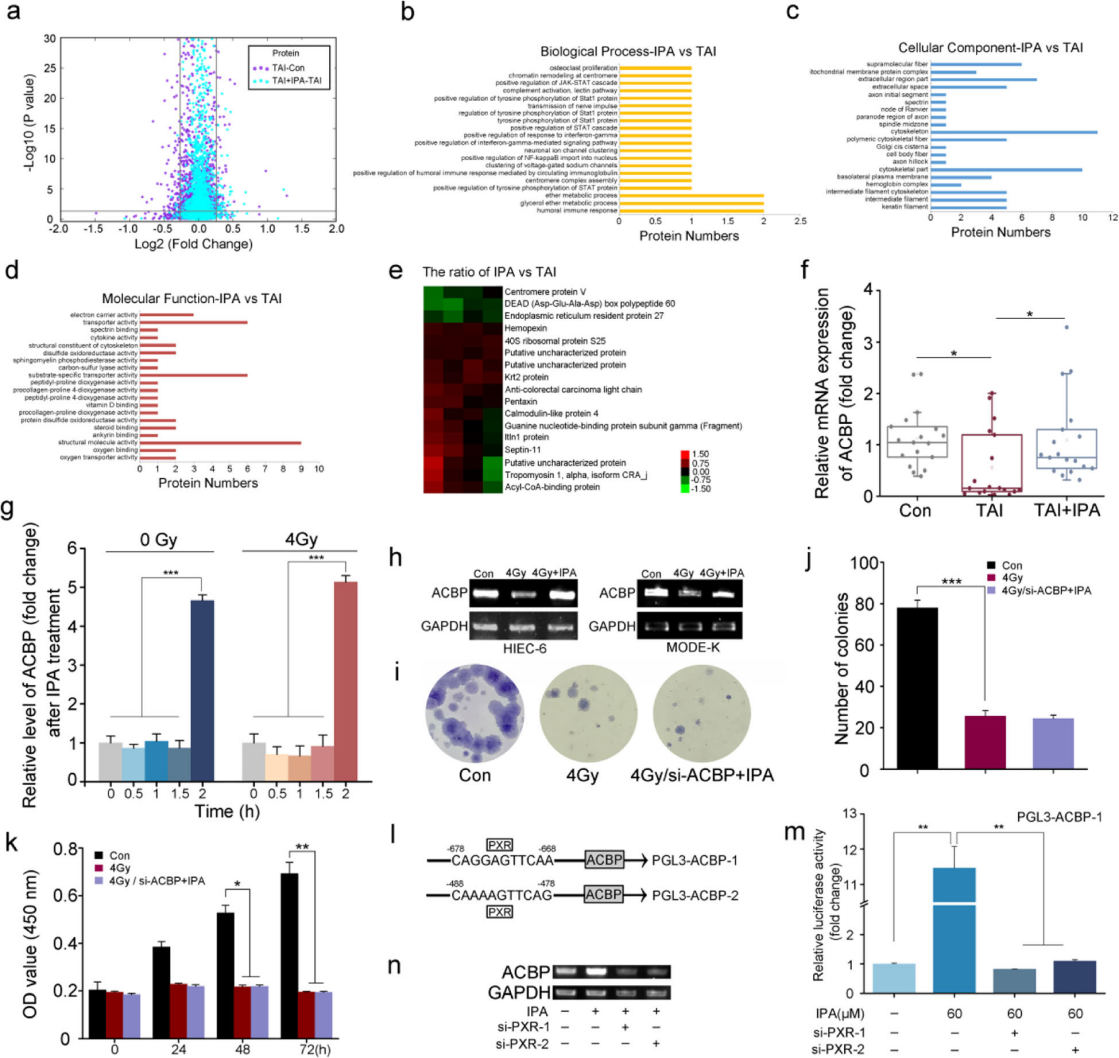
IPA replenishment reprograms small intestinal protein expression profile following TAI challenge. **a** Volcano plots of identified different proteins from the small intestine of mice with or without IPA treatment. In the volcano plots, each point represented a protein. **b**–**d** Bioinformatics analysis of different proteins in small intestine of mice in IPA gavage group compared to the TAI group through gene ontology (GO) in biological process (**b**), cellular component (**c**), and molecular function (**d**). Information on the number of involved proteins in a term is shown on the *x*-axis. **e** Hierarchical cluster analysis for the different proteins in the small intestine of mice in IPA gavage group compared to TAI group. **f** The expression level of ACBP was examined in small intestine tissues from male mice by qRT-PCR. Mean ± SEM. Significant between each two cohort differences are indicated: *P < 0.05; Student’s *t* test, *n* = 18 per group. **g** The relative level of ACBP were measured at the time of 0, 0.5, 1, 1.5, and 2 h with (or without) 4 Gy irradiation after IPA treatment (37.8 μg/mL) by qRT-PCR. Mean ± SEM. Significant differences between each two cohorts are indicated: ***P < 0.005; Student’s *t* test. **h** The mRNA levels of ACBP were examined in HIEC-6 and MODE-K cells which included control, 4 Gy irradiation, and 4 Gy irradiation with IPA supplement. **i**–**k** The effects of IPA (37.8 μg/mL) on the proliferation of ACBP siRNA-treated HIEC-6 cells were assessed by cloning formation (**i**, **j**) and CCK-8 assays (**k**), respectively. Mean ± SEM. Significant differences between each two cohorts are indicated: *P < 0.05, **P < 0.01, and ***P < 0.005; Student’s *t* test. **l** A model showed the predicted binding site for PXR at 678–668 nt and 488–478 nt of ACBP mRNA promoter named PGL3-ACBP-1 and PGL3-ACBP-2. **m** The effect of PXR and IPA on PGL3-ACBP-1 reporter was measured by luciferase reporter gene assays in HIEC-6 cells. Mean ± SEM. Significant differences between each two cohorts are indicated: **P < 0.01; Student’s *t* test. **n** The expression of ACBP was examined by qRT-PCR after transfection of HIEC-6 cells with si-PXR and (or) treated with IPA (37.8 μg/mL)

To further explore the molecular mechanism of radioprotection by IPA, we interrogated the responses of irradiated hosts to IPA treatment. iTRAQ analysis was performed to identify small intestinal proteomic changes among control and TAI-exposed male mice with or without IPA replenishment. The significant differentially expressed proteins were detected through screening of the reliable proteins with a *P* value less than 0.05 and multiple changes greater than 1.2 or less than 0.83, from which 183 significant differential proteins of control contrast TAI and 62 significant differential proteins of TAI contrast IPA showed differential accumulation in these two comparisons. As shown in Fig. 6a, the differential protein expression patterns were illustrated through the volcano plot. Moreover, we analysis all of the differential proteins via gene ontology (GO) enrichment, which was classified into biological process, cellular component, and molecular function. The biological process analysis for the differential proteins in small intestine of TAI-challenged mice compared with control mice were shown in Additional file 1: Figure S8A, from which the differential proteins of small intestine were mainly attributed to organonitrogen compound metabolic process (14.8%), cellular amide metabolic process (9.4%) and peptide metabolic process (9.4%), whereas the differential proteins detected among IPA group compared to TAI group (Fig. 6b) were majoring in humoral immune response (8.7%), glycerol ether metabolic process (8.7%) and ether metabolic process (8.7%). Besides that, intracellular organelle part and cytoskeleton were the most representative terms in cellular component (Fig. 6c and Additional file 1: Figure S8B). Regarding the molecular function, 23.4% and 13% of the detected different proteins were annotated displaying the structural molecule activity or substrate-specific transporter activity in TAI group (Additional file 1: Figure S8C) or IPA supplement group (Fig. 6d), respectively. On the basis of proteomic changes, we found that irradiation decreased the level of acyl-CoA-bind protein (ACBP) from the small intestinal tissues while IPA replenishment reversed the change through iTRAQ proteomic method and quantitative real-time PCR in male and female mice (Fig. 6e, f and Additional file 1: Figure S8D, E). In addition, ACBP was activated by IPA in 2 h with or without irradiation exposure in normal human intestinal epithelial cells (HIEC-6) (Fig. 6g), implying that ACBP might be a rapid response gene for rehabilitation drove by IPA.

Next, HIEC-6 and mouse intestinal epithelial cells (MODE-K) were employed to further validate the role of ACBP in IPA-mitigated radiation mortality. As shown in Fig. 6h and Additional file 1: Figure S9A, IPA treatment erased the reduction of ACBP following irradiation. Using special siRNA targeting ACBP (Additional file 1: Figure S9B), cloning formation and CCK-8 assays further revealed that depletion of ACBP blocked the protective function of IPA toward irradiation in HIEC-6 cells (Fig. 6i–k). IPA has been reported as a ligand for pregnane X receptor (PXR) [32]. Bioinformatics analysis showed two PXR binding sites located in the region of ACBP promoter (http://alggen.lsi.upc.es/). Thus, two fragments carried the binding sites were constructed into PGL3-basic plasmid and named as PGL3-ACBP-1 and PGL3-ACBP-2 (Fig. 6l). Luciferase reporter assays revealed that IPA activated the ACBP promoter carrying PXR binding site region (position 668–678; Fig. 6m), rather than the other region (position 478–488; Additional file 1: Figure S9C). Notably, using special siRNA to silence PXR (Additional file 1: Figure S9D) abrogated the activation of PGL3-ACBP-1 and the upregulation of ACBP drove by IPA in HIEC-6 cells (Fig. 6m, n). Together, our observations indicate that the protective function of IPA against irradiation partly depends on ACBP.

### ACBP contributes to the radioprotection of IPA via PXR

**Fig. 7:**
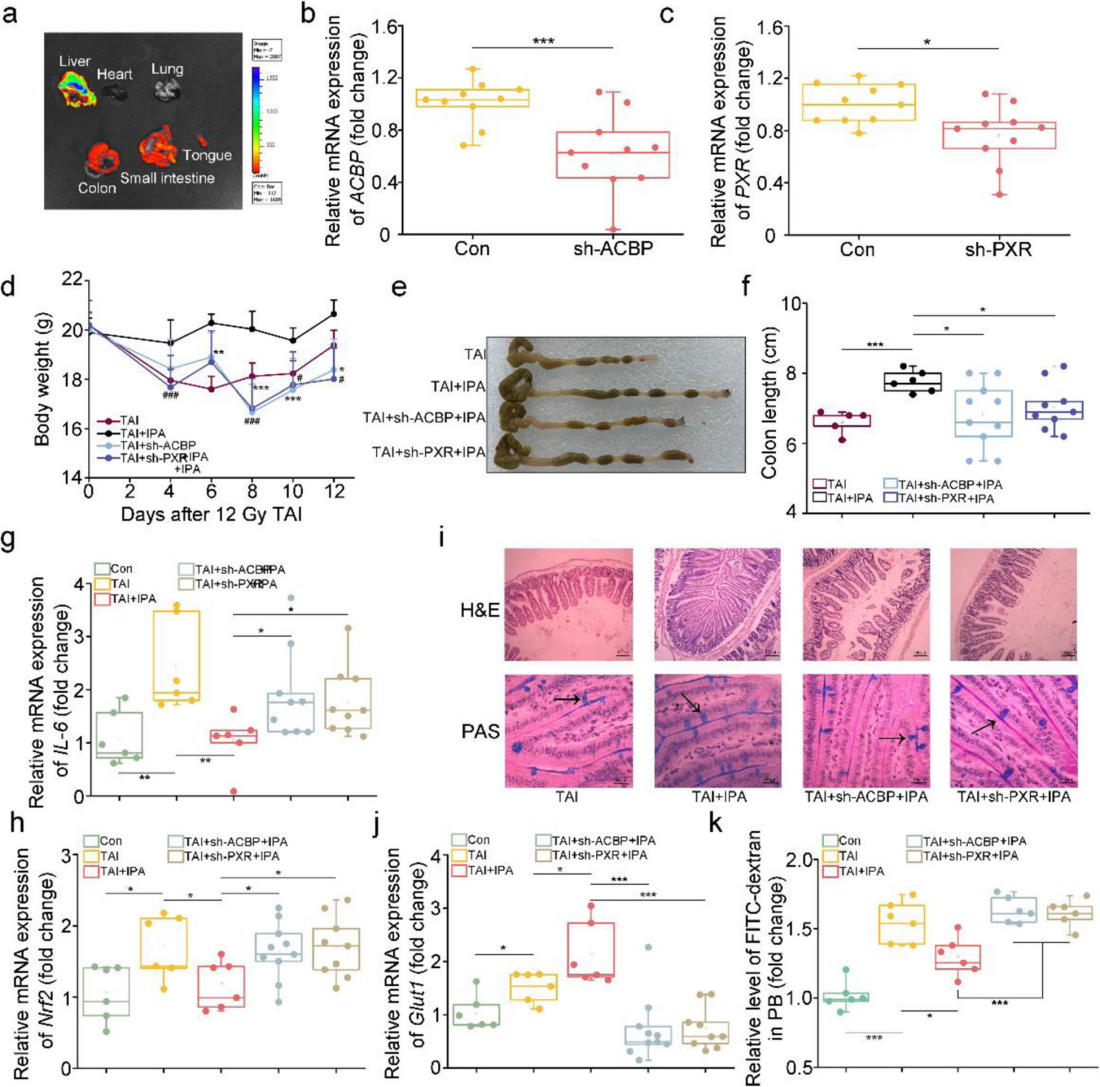
ACBP contributes to the protective function of IPA toward irradiation via PXR. **a** The presence of gene expression after injection in various organs, including the liver, heart, lung, colon, small intestine, and tongue, as confirmed by bioluminescent imaging. **b**, **c** The expression level of ACBP (**b**) and PXR (**c**) was examined in the small intestine tissues by qRT-PCR. Significant differences are indicated: *P < 0.05 and ***P < 0.005; Mean ± SEM. Student’s *t* test, *n* = 10 per group. **d** Body weights were compared among four group mice after 12Gy TAI, *n* = 18 per group; *P < 0.05, **P < 0.01, and ***P < 0.005 represent TAI + sh-ACBP group compared with TAI + IPA group; ^#^*P* < 0.05, ^##^*P* < 0.01, and ^###^*P* < 0.005 represent TAI + sh-PXR group compared with TAI + IPA group; Student’s *t* test. **e**, **f** Photographs (**e**) and length (**f**) of dissected colon from mice in the four groups, the colon tissues were obtained at day 21 after 12 Gy TAI. Mean ± SEM. Significant differences between each two cohorts are indicated: *P < 0.05 and ***P < 0.005; Student’s *t* test, *n* = 5 for TAI group, *n* = 6 for TAI + IPA group, *n* = 10 for TAI + sh-ACBP group, *n* = 9 for TAI + sh-PXR group. **g**, **h**, **j** The expression levels of *IL-6* (**g**), *Nrf2* (**h**), and *Glut1* (**j**) were examined in the small intestine tissues by qRT-PCR. The small intestine tissues were obtained at day 21 after 12 Gy TAI. Mean ± SEM. Significant differences between each two cohorts are indicated: *P < 0.05, **P < 0.01, and ***P < 0.005; Student’s *t* test, *n* = 6 for control group, *n* = 6 for TAI group, *n* = 6 for TAI + IPA group, *n* = 10 for TAI + sh-ACBP group, *n* = 9 for TAI + sh-PXR group. **i** The morphology of the small intestine was shown by H&E (×100 magnification; Scale bar: 100 μm) and PAS (×1000 magnification; Scale bar: 50 μm) staining. The small intestine tissues were obtained at day 21 after 12 Gy TAI. The arrows point to the goblet cells. **k** The FITC-dextran in PB was assessed at day 21 after 12 Gy TAI. Mean ± SEM. Significant differences between each two cohorts are indicated: **P < 0.01 and ***P < 0.005; Student’s *t* test, *n* = 6 for control group, *n* = 6 for TAI group, *n* = 6 for TAI + IPA group, *n* = 10 for TAI + sh-ACBP group, *n* = 9 for TAI + sh-PXR group

Hydrodynamic-based gene delivery has emerged as an efficient and simple method for the intracellular transfection of plasmid in vivo [33]. To silence the expression of ACBP (or PXR), the specific shRNA targeting ACBP (or PXR) was cloned in pRNA-U6.1/Neo plasmids and rapidly injected into retro-orbital sinus of male mice to inhibit the expression of ACBP (or PXR). Fluorescence imaging validated the accumulation of the reconstructive pRNA-U6.1/Neo plasmids in various dissected organs including the liver, colon, small intestine, and tongue, but not in the heart and kidney (Fig. 7a). qRT-PCR further revealed the downregulation of ACBP (or PXR) expression in the small intestine following the special pRNA-U6.1/Neo plasmids injection (Fig. 7b, c), indicating that hydrodynamic-based retro-orbital sinus injection can serve as a means of introducing transgenes specifically to the small intestine. Then, we identified whether IPA replenishment accompanied with ACBP or PXR knockdown could still protect against ARS. After exposure to 12 Gy TAI, ACBP, or PXR silencing amplified the body weight loss (Fig. 7d). Besides that, the irradiated mice with ACBP or PXR deletion showed deteriorative GI tract injuries characterized by shorter colon length (Fig. 7e, f), higher inflammation and ROS levels (Fig. 7g, h and Additional file 1: Figure S10A), fewer intestinal villi and goblet cells (Fig. 7i), worsen the epithelial integrity (Fig. 7j and Additional file 1: Figure S10B, C), and GI tract barrier function (Fig. 7k), indicating that IPA performs radioprotection to GI tract partly depending on PXR/ACBP signaling.

## Discussion

Recent efforts to define the complex nature of diseases have focused on the contribution of host microbiota. Fecal microbiota transplantation (FMT) is historically known to be a therapeutic intervention to treat GI and non-GI diseases, covering CDI [34] and cancers [35]. Recently, we reported that FMT might be employed as a therapeutic avenue to protect against ARS. In clinical application, however, FMT has some limitations including aesthetic concerns, costs of donor screening, and material preparation and administration [36, 37]. Hence, search for effective treatment options through identifying the functional constituents in fecal pellets superseding FMT to prevent various diseases may have broader implications. The components in fecal samples include viable bacteria (~ 1011 per gram of wet stool), colonocytes (~ 107 per gram of wet stool), archaea (~ 108 per gram of wet stool), viruses (~ 108 per gram of wet stool), fungi (~ 106 per gram of wet stool), protists, and metabolites [38]. Once established in the intestine, the microbiota influences host immune response [39] and gastrointestinal barrier function [32] through metabolic activities such as short-chain fatty acids from carbohydrate metabolism and tryptophan metabolites from amino acid metabolism. In light of our previous study, we focused on the alteration of gut microbiota metabolites, and obtained that radiation exposure lessened the level of IPA in fecal pellets, which could be preserved by FMT.

These microbial metabolites are generated through microorganism-microorganism and host-microorganism interactions, and there is a growing appreciation of a role for this co-metabolism in human health and disease [40, 41]. There is an emerging understanding the relationship between microbiota metabolism and host physiology, which includes host metabolism [42, 43], gut immunity [44], cancer [45], asthma [46], and nervous system [47]. Moreover, some studies exist that IPA, a bacterial-mediated production of bioactive indole-containing metabolites derived from tryptophan, is a ligand for PXR and promotes intestinal barrier integrity through downregulation of epithelial TNFɑ, induction of MDR1, and regulation of epithelial junctional complexes [32]. The intestinal epithelial is one of the most rapidly renewing system in animal, which makes the small intestine as the most sensitive and vulnerable part of GI tract to irradiation [48]. Specifically, radiation stimuli impairs enteric integrity and mediates intestinal barrier dysfunction [29]. Here, we observed that oral gavage of IPA enhanced the integrity and function of GI tract. Recently, several studies have shown that microbiota-derived indole metabolites promote human and murine intestinal homeostasis through mitigating inflammatory responses like interleukin-10 receptor [20, 49]. In this study, radiation challenge elevated the levels of inflammatory mediators, such as IL-6 and TNFɑ, in PB, and intestine tissues; however, IPA replenishment by oral route erased the alterations. Thus, IPA restores radiation-induced intestinal flora dysbiosis might through regulating inflammatory responses which need further study. In addition, it is well documented that IPA protects neurons from ischemia-induced neuronal damage by reducing DNA damage and lipid peroxidation [50]. Intriguingly, IPA replenishment restored the size and weight of the spleen (and thymus) and raised a series of blood cell counts, indicating the rehabilitation of hematopoietic toxicity concomitant with total body irradiation. Together, irradiated mice that received IPA not only exhibited fewer mortality and radiation induced physical signs, but also had significantly less damage to GI tract and hematopoietic system. Importantly, IPA performed radioprotection to both male and female experimental animals and did not precipitate cancer cell proliferation in tumor-bearing mouse models. Given IPA is a metabolite of gut microbiota emerged in GI tract, our findings underpin that IPA might be employed as a safe remedy to protect against hematopoietic and GI toxicity intertwined with radiation exposure in pre-clinical settings.

To date, except for bile acids, the effects of gut microbiota metabolites on the enteric bacteria remain poorly defined [51]. In the previous study, we observed that TAI exposure decreased the abundance of intestinal *g_Clostridium*. However, IPA replenishment abolished the shifts (data not shown). Given the relationship between g_*Clostridium* and IPA production [28], we further tested IPA content in feces pellets which appeared IPA treatment increased TAI-induced lower content of IPA (data not shown). Accordingly, we supposed that IPA exerted its protective effects to irradiation partly dependent on the enteric microbes. To address this hypothesis, mice were domesticated with antibiotics. As expected, ABX-challenged mice failed to response to IPA treatment, represented as serious GI tract toxicity following TAI exposure. The findings suggest that gut microbiome is indispensable parameters for the function of microbiota metabolites. In addition, our observations revealed that oral gavage of IPA changed irradiation-shifted intestinal bacterial structure. Owing to the relationship between gut microbiota and hosts’ radiosensitivity [52], the protective function against irradiation of IPA might partly dependent on the alterations of gut microbiota. Gene expression profile is accounted for the fate of cells [53, 54]. Thus, we further obtained that IPA administration reprogrammed the protein expression profile of small intestine tissues in irradiated mice. Specifically, our data supported that ACBP was an essential regulator responding to radiation-caused GI injury. ACBP has been shown to transport acyl-CoAs and donate them to various metabolic pathways [55], influence directly glucose-induced insulin secretion, stimulation of steroidogenesis, and modulation of cell proliferation [22]. Further, a study shows that ACBP is required for maintaining normal epidermal barrier function [56]. Patients with HIV infection reveal an inverse correlation of serum IPA and LPS (marker of intestinal microbial translocation), demonstrating that IPA regulates intestinal permeability in humans [57]. In the present study, IPA treatment recovered radiation lessened ACBP via orphan receptor PXR and showed beneficial effects on intestinal epithelial barrier function, which consistent with the notion that altered microbial metabolites correlates with intestinal homeostasis and intestinal barrier function. In addition, we used hydrodynamic-based gene delivery technique to block the expression of ACBP or PXR, which lost the beneficial effects of IPA administration on intestinal homeostasis and intestinal barrier function. These findings furtherly proved that PXR/ACBP signaling were essential to IPA exerted protective effects. Activation of PXR inhibits tumorigenicity of colon cancer cells [58], and PXR agonists may have potentials in inhibiting inflammation related diseases [59]; besides, we also identified that IPA did not precipitate tumor growth in vivo, indicating that IPA might be employed in clinical settings without potential pitfalls. Thus, our work defines that indole metabolites IPA could be employed as a supportive therapy in individuals with ARS as well as provides important biology steps toward a more comprehensive understanding of gut microbiota and its metabolites.

## Conclusions

In the present study, we identify gut microbiota metabolite indole-3-propionic acid (IPA) protects against radiation-associated hematopoietic syndrome and GI syndrome without accelerating tumor growth. Mechanistically, IPA retains enteric bacterial configurations and small intestinal protein profile of radiation-challenged hosts. In addition, IPA activates enteric PXR/ACBP signaling to perform protective function toward GI toxicity. Clinically, IPA might be employed as a microbiome-based therapeutic approach toward radioactive disease, and cancer patients might replenish IPA to alleviate clinical complications after radiotherapy. Importantly, ACBP is a novel target for the development of radioprotective drugs.

## Methods

### Mice

Six- to 8-week-old-male (around 20 g)/female (around 18 g) C57BL/6J mice and 4-week-old-male/female BALB/c athymic nude mice were purchased from the Beijing Huafukang Bioscience Co., Inc. (Beijing, China). Mice were housed in the Specific Pathogen Free (SPF) level animal facility at the Institute of Radiation Medicine (IRM), the Chinese Academy of Medical Sciences (CAMS). Mice were kept under standard conditions (ambient temperature 22 ± 2 °C, air humidity 40–70% and a 12/12-h light/dark cycle) and continuous access to a standard diet and water. Animal experiments were performed according to the institutional guidelines approved by the Animal Care and Ethics Committee of IRM-PUMC, which complied with the Guide for the Care and Use of Laboratory Animals and the National Institutes of Health guide for the Care and Use of Laboratory Animals.

### Cell culture

HIEC-6, MODE-K, HCT-8, and ME-180 cells were obtained from the American Type Culture Collection (ATCC) and certified to be mycoplasma-free. The passage numbers of those cell lines during the experimental period were no more than eight. The cells were cultured with 10% fetal bovine serum (Gibco, Grand Island, NY, USA), 100 U/ml penicillin and 100 mg/ml streptomycin and grown at 5% CO_2_ and 37 °C.

### Irradiation study

A Gammacell-40 137Cs irradiator (Atomic Energy of Canada Limited, Chalk River, ON, Canada) at a dose rate of 1.0 Gy per minute was used for all experiments. Mice treated with total body irradiation (TBI) were exposed to 4 Gy (for hematopoietic system experiments) or 7.2 Gy (for survival rate experiments) γ-ray. Mice were anesthetized with 3.5% chloral hydrate intraperitoneal injection (around 200 μL per mouse) and treated with total abdominal irradiation (TAI) for GI tract experiments were exposed to 12 Gy γ-ray using a lead shielding (Additional file 1: Figure S11) so that the whole abdomen will be irradiated and the other parts of the mouse will be shielded. Control mice were sham-irradiated.

### Experimental group

(1) The control group is the following: healthy 6- to 8-week-old-male or female C57BL/6 mice. (2) TBI group: in survival rate test, mice were exposed to 7.2 Gy total body irradiation, and in hematopoietic system experiments, mice were exposed to 4 Gy total body irradiation. (3) TAI group: Mice were exposed to 12 Gy total of abdominal irradiation. (4) TBI + IPA group: Mice were treated with IPA 7.5 mg/ml IPA through oral route, dissolved in sterile water in 0.2 ml volume per mice for 15 consecutive days after 4 Gy or 7.2 Gy TBI. (5) TAI + IPA group: Mice were treated with IPA 7.5 mg/ml IPA through oral route, dissolved in sterile water in 0.2 ml volume per mice for 15 consecutive days after 12 Gy TAI. (6) TAI + ABX + IPA group: Mice were treated for 20 days with antibiotics (ABX) in their drinking water before irradiation. Then, mice were treated with IPA 7.5 mg/ml IPA through oral route and dissolved in sterile water in 0.2 ml volume per mice for 15 consecutive days after 12 Gy TAI. (7) TAI + sh-ACBP+IPA group: After 12 Gy TAI mice were immediately injected with sh-ACBP plasmid solution, then treated with IPA 7.5 mg/ml IPA through oral route, and dissolved in sterile water in 0.2 ml volume per mice for 15 consecutive days. (8) TAI + sh-PXR + IPA group: After 12 Gy TAI mice were immediately injected with sh-PXR plasmid solution, then treated with IPA 7.5 mg/ml IPA through oral route, and dissolved in sterile water in 0.2 ml volume per mice for 15 consecutive days.

### Quantification of IPA

Fecal microbiota transplantation was performed to mice with total body or abdominal irradiation for 10 days. Then, the fecal pellets were collected under SPF conditions. The fecal pellets from each cohort was weighed and diluted with 1 ml of saline per 0.1 g of stool. Briefly, the stool was steeped in saline for about 15 mins, shaken and then centrifuged at 800 rpm for 3 mins. The supernatant was obtained to assess the level of IPA using ELISA kit according to the manufacturer's protocol (Zcibio, Shanghai, China). Optical density was read at 450 nm (Rayto, Shenzhen, China).

### Antibiotics test

TAI + ABX and TAI + ABX + IPA mice were treated for 20 days with Ciprofloxacin (125 mg/L, Sigma-Aldrich, Madrid, Spain), Metronidazole (100 mg/L, Sigma-Aldrich, Madrid, Spain), Vancomycin (50 mg/L, Sigma-Aldrich, Madrid, Spain), Streptomycin (100 U/L, Solarbio, Beijing, China) and Penicillin (100 U/L, Solarbio, Beijing, China) in their drinking water before irradiation, respectively. The fresh antibiotic solution was prepared every day to promise its activity.

### Histology

Following euthanasia, the small intestines of mice were fixed in 4% buffered formalin overnight at room temperature and then embedded in paraffin. Tissues were sectioned at 5 μm thickness and dipped in hematoxylin and eosin (H&E) using standard protocols. For PAS staining, the small intestines of mice were fixed in Carnoy's solution. Dewaxed sections were hydrated and incubated in 1% periodic acid for 10 min followed by incubation in Schiff’s reagent for 10 min. Sections were counterstained with Mayer’s hematoxylin for 30 s, washed and dehydrated before mounting with Pertex. For immune-histochemical staining (IHC), deparaffinized sections were rehydrated and stained using the primary antibodies of mouse anti-Ki-67 (Cell Signaling Technology, MA, USA, 1:400 dilution). Then horseradish peroxidase (HRP) - conjugated secondary antibody (ZSGB-BIO, Beijing, China) was used. For HRP-conjugated secondary antibody, stained by 3, 3′-diaminobenzidine Staining Kit (BD Biosciences), followed by hematoxylin nuclear counterstaining.

### Untargeted metabolomics—metabolite extraction

Feces were individually grounded with liquid nitrogen and the homogenate was suspended with prechilled 80% methanol and 0.1% formic acid by well vortexing. The samples were incubated on ice for 5 min and then were centrifuged at 15000 rpm, 4 °C for 5 min. A some of supernatant was diluted to final concentration containing 60% methanol by LC-MS grade water. The samples were subsequently transferred to a fresh Eppendorf tube with 0.22 μm filter and then were centrifuged at 15000 g, 4 °C for 10 min. Finally, the filtrate was injected into the LC-MS/MS system analysis.

### Untargeted metabolomics—UHPLC-MS/MS analysis

LC-MS/MS analyses were performed using a Vanquish UHPLC system (Thermo Fisher) coupled with an Orbitrap Q Exactive HF-X mass spectrometer (Thermo Fisher). Samples were injected onto an Hyperil Gold column (100 × 2.1 mm, 1.9 μm) using a 16-min linear gradient at a flow rate of 0.2 mL/min. The eluents for the positive polarity mode were eluent A (0.1% FA in Water) and eluent B (Methanol). The eluents for the negative polarity mode were eluent A (5 mM ammonium acetate, pH 9.0) and eluent B (Methanol). The solvent gradient was set as follows: 2% B, 1.5 min; 2-100 % B, 12.0 min; 100 % B, 14.0 min; 100-2 % B, 14.1 min; 2 % B, 16 min. Q Exactive HF-X mass spectrometer was operated in positive/negative polarity mode with spray voltage of 3.2 kV, capillary temperature of 320 °C, sheath gas flow rate of 35 arb and aux gas flow rate of 10 arb.

### Untargeted metabolomics—data analysis

The raw data files generated by UHPLC-MS/MS were processed using the Compound Discoverer 3.0 (CD 3.0, Thermo Fisher) to perform peak alignment, peak picking, and quantitation for each metabolite. The main parameters were set as follows: retention time tolerance, 0.2 minutes; actual mass tolerance, 5 ppm; signal intensity tolerance, 30%; signal/noise ratio, 3; and minimum intensity, 100000. After that, peak intensities were normalized to the total spectral intensity. The normalized data was used to predict the molecular formula based on additive ions, molecular ion peaks and fragment ions. And then peaks were matched with the mzCloud (https://www.mzcloud.org/) and ChemSpider (http://www.chemspider.com/) database to obtain the accurate qualitative and relative quantitative results.

### Bacterial diversity analysis

Stool samples were freshly collected from two independent experiments and stored at -80 °C until use. DNA was extracted from the stool using the Power Fecal® DNA Isolation Kit (MoBio Carlsbad, CA USA). The DNA was recovered with 30 ml of buffer in the kit. PCR products were mixed in equidensity ratios. Then, mixture PCR products were purified with Qiagen Gel Extraction Kit (Qiagen, Germany). The 16S ribosomal RNA (rRNA) V4 was amplified used specific primer. All PCR reactions were carried out with Phusion® High-Fidelity PCR Master Mix (New England Biolabs). Sequencing libraries were generated usingTruSeq® DNA PCR-Free Sample Preparation Kit (Illumina, USA) following manufacturer's recommendations and index codes were added. The library quality was assessed on the Qubit@ 2.0 Fluorometer (Thermo Scientific) and Agilent Bioanalyzer 2100 system. At last, the library was sequenced on an IlluminaHiSeq2500 platform and 250 bp paired-end reads were generated. Paired-end reads was assigned to samples based on their unique barcode and truncated by cutting off the barcode and primer sequence. Paired-end reads were merged using FLASH (V1.2.7,http://ccb.jhu.edu/software/FLASH/), a very fast and accurate analysis tool, which was designed to merge paired-end reads when at least some of the reads overlap the read generated from the opposite end of the same DNA fragment, and the splicing sequences were called raw tags. Quality filtering on the raw tags were performed under specific filtering conditions to obtain the high-quality clean tags according to the QIIME (V1.7.0, http://qiime.org/index.html) quality-controlled process. The tags were compared with the reference database (Gold database, http://drive5.com/uchime/uchime_download.html) using UCHIME algorithm (UCHIME Algorithm, http://www.drive5.com/usearch/manual/uchime_algo.html) to detect chimera sequences, and then the chimera sequences were removed. Then the Effective Tags finally obtained. (Novogene Bioinformatics Technology Co., Ltd.). Sequences analysis was performed by Uparse software (Uparse v7.0.1001, http://drive5.com/uparse/). Sequences with ≥97% similarity were assigned to the same OTUs. Representative sequence for each OTU was screened for further annotation. For each representative sequence, the Silva123 Database was used based on RDP classifier (Version 2.2, http://sourceforge.net/projects/rdpclassifier/) algorithmto annotate taxonomic information. Briefly, each cohort contains 12 mice, and 6 mice share one cage. For gut microbiota analysis, we collected 2 fecal pellets from one cage and 3 from the other cage to avoid cage effects. Six C57BL/6 J mice without irradiation were grouped as Cond 6/12 at day 6/12. Six mice with 12 Gy TAI at day 6/12 were grouped as TAI d 6/12. For IPA performance, six mice have been treated with IPA for 6/12 days, their stool samples were collected and grouped as TAI + IPA d 6/12. The primers are listed in Supplementary Table 1.

### Peptide fractionation and identification by MS/MS

In each group of mice, we randomly pooled tissue samples from 3, 3, and 4 mice to generate three protein extracts 1, 2, 3, respectively. The protein extraction method was in accordance with the manufacturer’s protocol. The tryptic peptides were labeled by the 8-plex iTRAQ reagents (AB Sciex, FosterCity, CA) according to the manufacturer’s protocol. After 2 h labeling reactions, the labeled peptides were pooled together for further peptide fractionation and identification. Each pool of mixed peptides was lyophilized and dissolved in solution A (25% ACN and 10 mM KH2PO4, pH = 3). Then, they were loaded onto an RP column (Luna C18, 4.6 × 150 mm, Phenomenex, CA) and eluted by a step linear elution program: 0-10 min equilibrated in 100 % solution A, 10-15 min fast elution from 0 to 12% of solution B (25% ACN, 2 M KCL, 10 mM KH2PO4, pH = 3), 15-45 min linear elution from 12 to 56% of solution B, and 45-57 min washing elution from 56 to 100 % of solution B. The RP HPLC procedures were manipulated in an LC solution 20A (Shimadzu, Nakagyo-ku, Kyoto, Japan) with a flow rate of 0.5 mL/min, and the peptides were monitored at 214 nm. The fractionated peptides were collected at one tube/min during the linear elution period and further pooled into the indicated fractions (10 for exosome, 15 for exosome-free, and 18 for cells). Each fraction was analyzed by a Q-Exactive mass spectrometer (Thermo Fisher Scientific) coupled with an Easy-nLC 1000 UPLC (Thermo Fisher Scientific) system twice. Peptides were loaded on a precolumn (10 μm-C18 resin, 75 μm × 8 cm) and separated with an analytical column (3-μm C18 resin, 75 μm × 11 cm, YMC Co., Ltd.) using acetonitrile gradients from 5 to 40% in 65 min at a flow rate of 400 nL/min. Spectra were acquired in data-dependent mode. The 10 most intense ions of +2, +3, and + 4 charge from each full scan (R = 70000) were isolated for HCD MS2 (R = 17500) at 27% normalized collision energy (NCE) with a dynamic exclusion time of 150 s.

### Database searches for peptide, protein identification, and quantitative data analysis

The raw MS/MS data were converted to MGF format by Proteome Discoverer 1.3 (Thermo Fisher Scientific, Waltham, MA), and the exported MGF files were searched by Mascot 2.3 (Matrix Science, Boston, MA) against the database Uniprot (selected for Mus., unknown version, 16700 entries). An automatic decoy database search was performed. Several parameters in Mascot were set for peptide searching, including iTRAQ 8-plex for quantification, tolerance of one missed cleavage of trypsin, methylthio for cysteine as a fixed modification, and oxidation for methionine as a variable modification. The precursor mass tolerance was 15 ppm, and the product ion tolerance was 0.8 Da. After database searching, the DAT files were imported into Scaffold v4.3.2 (Proteome Software Inc., Portland, OR). Scaffold was used to organize all data, quantitate proteins, and validate peptide identification using the Peptide Prophet algorithm, and unique proteins with at least two unique peptides with a false discovery rate (FDR) < 0.01 were qualified for further quantification analysis. The fold changes in protein abundance were defined as the median ratio of all significantly matched spectra with tag signals. For iBAQ intensity analysis through Maxquant software (version 1.5.0), proteins identified by at least 2 unique peptides were used. Heat maps of function categories and abundance changes of the differential proteins were generated by R software.

### In vivo tumor xenograft assay

Four-week-old-male and female BALB/c athymic nude mice were housed and treated according to the guidelines established by the National Institutes of Health Guide for the Care and Use of Laboratory Animals. Briefly, HCT-8 (or ME-180) cells were harvested and suspended at 2 × 10^7^ cells per mL in sterile PBS. Groups of 4-week-old-male (injected with HCT-8 cells) and female (injected with ME-180 cells) nude mice were subcutaneously injected at the shoulder with 0.2 mL of the cell suspensions. Once the tumors reached an average volume of 100 mm^3^, the mice were randomly divided into four groups (*n* = 7 per group) and respective treatments were given. Group I (vehicle control): saline as control; Group II: IPA; Group III: local irradiation; Group IV: IPA + local irradiation. IPA (7.5 mg/ml) was given to tumor-bearing mice by oral gavage before each dose of irradiation. Fractionated irradiation treatment (3 Gy per day) was given for days, till a cumulative dose of 12 Gy was achieved. For the radiation, mice were positioned under a lead shield (Additional file 1: Figure S11) so that only the tumor area was exposed. Tumor sizes were monitored twice weekly using digital caliper. Tumor volume (*V*) was monitored by measuring the length (*L*) and width (*W*) of the tumors and was calculated using the formula (*L* × *W*^2^) × 0.5. After 25 days, tumor-bearing mice were sacrificed.

### Statistical analysis

Each experiment was repeated at least three times. Data were assessed normal distribution using the Kolmogorov–Smirnov test. The data are presented as the means ± SEM with respect to the number of samples (*n*) in each group. Significance was assessed by comparing the mean values using Student’s *t* test and Wilcoxon rank sum test for independent groups as follows: *P < 0.05; **P < 0.01; ***P < 0.005. Kaplan-Meier analysis was performed for survival analysis, and significance between survival curves was determined by a log rank test. Results with *P* < 0.05 were considered statistically significant. Experiments through the study have been performed at least three times.

## Supplementary information

**Additional file 1: Table S1.** List of primers used in this paper. Figure S1. IPA facilitated the proliferation of irradiated HIEC-6 cells and MODE-K cells. Figure S2. Oral gavage of IPA ameliorates TBI-associated hematopoietic system injury. Figure S3. IPA administration improves GI tract function after total abdominal irradiation. Figure 4. IPA protects against radiation toxicity in mouse models without accelerating tumor growth. Figure 5. IPA treatment changes irradiation-shaped intestinal bacterial structure at day 6 after TAI. Figure 6. IPA preserves irradiation-shifted enteric bacterial composition at day 12 after TAI. Figure 7. Impact of antibiotics (ABX) and IPA on GI tract function after total abdominal irradiation. Figure 8. TAI changes the protein expression profile of small intestine. Figure 9. ACBP contributes to the protective function of IPA toward irradiation *via* PXR. Figure 10. ACBP or PXR inhibition blocked the protective function of IPA toward irradiation. Figure 11. Photographs of the lead shielding apparatus used in this study. The left photograph showed the lead shielding without lid, and the right photograph showed the lead shielding with lid

## Data Availability

Raw 16S rRNA gene sequences for all samples used in this study have been deposited in European Nucleotide Archive under project accession no. PRJEB34265.

## Abbreviations

ABX: Antibiotics
ACBP: Acyl-CoA-binding protein
ARS: Acute radiation syndrome
CCK-8: Cell counting kit-8
DBI: Diazepam-binding inhibitor
FMT: Fecal microbiota transplantation
GI: Gastrointestinal
GO: Gene ontology
HGB: Hemoglobin
HSPCs: Hematopoietic stem and progenitor cell
IPA: Indole 3-propionic acid
LEfSe: Linear discriminant analysis effect size
LY: Lymphocytes
NE: Neutrophil granulocytes
NMDS: Non-metric multidimensional scaling
PB: Peripheral blood
PCA: Principal component analysis
PCoA: Principle coordinate analysis
PXR: Pregnane X receptor
RBC: Red blood cell
TAI: Total abdominal irradiation
TBI: Total body irradiation
WBC: White blood cell
