# Supplementary material for: Gut microbiota-derived indole 3-propionic acid protects against radiation toxicity via retaining acyl-CoA-binding protein
Source: Microbiome. 2020 May 20;8:69. doi: 10.1186/s40168-020-00845-6 (PMC7241002; doi:10.1186/s40168-020-00845-6)
Supplement: Supplementary file 2 — Additional file 1: Table S1. List of primers used in this paper. Figure S1. IPA facilitated the proliferation of irradiated HIEC-6 cells and MODE-K cells. Figure S2. Oral gavage of IPA ameliorates TBI-associated hematopoietic system injury. Figure S3. IPA administration improves GI tract function after total abdominal irradiation. Figure 4. IPA protects against radiation toxicity in mouse models without accelerating tumor growth. Figure 5. IPA treatment changes irradiation-shaped intestinal bacterial structure at day 6 after TAI. Figure 6. IPA preserves irradiation-shifted enteric bacterial composition at day 12 after TAI. Figure 7. Impact of antibiotics (ABX) and IPA on GI tract function after total abdominal irradiation. Figure 8. TAI changes the protein expression profile of small intestine. Figure 9. ACBP contributes to the protective function of IPA toward irradiation via PXR. Figure 10. ACBP or PXR inhibition blocked the protective function of IPA toward irradiation. Figure 11. Photographs of the lead shielding apparatus used in this study. The left photograph showed the lead shielding without lid, and the right photograph showed the lead shielding with lid [file 40168_2020_845_MOESM1_ESM.docx]

**Supplementary information**

**Supplementary Methods**

**Oral and culture dosing of IPA**

C57BL/6J mice were treated with 3.75 or 7.5 mg/ml IPA through oral route, dissolved in sterile water in 0.2 ml volume per mice for 15 consecutive days. Cytological experiment used a series of concentrations of IPA dissolved in alcohol. The dose of IPA was kept through the cytological experiment.

**Antibodies**

Biotin-conjugated anti-mouse CD4 (clone GK1.5), anti-mouse CD8 (clone53-6.7), anti-mouse CD45R/B220 (cloneRA3-6B2), anti-mouse Ly6G/Gr-1 (clone RB6-8C5), anti-mouse CD11b (clone M1/70), anti-mouse Ter-119 (clone Ter-119), APC (clone 2B8), and anti-mouse Ly-6 A/E (Sca1) PE (clone D7), were obtained from eBioscience (San Diego, CA, USA). PerCP streptavidin was obtained from Biolegend (San Diego, CA, USA).

**Cloning formation assays**

For clonogenicity analysis, 48 h after transfection (or irradiation), about 500 viable cells were placed in 6-well plates and maintained in complete medium for about 3 weeks. Colonies were fixed with methanol and stained with methylene blue.

**Cell counting kit-8 assay**

The cell proliferative effects of IPA were assessed by CCK-8 assay according to the manufacturer's guidelines. Briefly, the cells were subsequently treated with various concentrations of IPA. Ten µl of CCK-8 reagents were added to assess cell proliferation every day from the first day until the third day.

**Plasmid constructions**

Two 500-600 base pair fragments of ACBP promoter were amplified from genome of HIEC-6 cells and then cloned into PGL3-Basic vector. The resulting vectors were sequenced and named PGL3-ACBP-1 and PGL3-ACBP-2. Sh-ACBP and sh-PXR sequences were forecasted by BLOCK-iT™ RNAi Designer, and then inserted into the pRNA-U6.1/Neo vector at BamH I and Hind III sites to obtain the plasmids. The plasmids were synthetized by GENEWIZ^®^ (Suzhou, China). All primers and the sequencing of sh-ACBP and sh-PXR were showed in Supplementary table 1.

**Cell transfection**

For cell transfection, the cells were cultured in a 6-well or 24-well plates for 24 h and then were transfected with siRNA. All transfections were performed using PEI (Sigma-Aldrich, Madrid, Spain) according to the manufacturer's protocol. The small interfering RNA targeting ACBP siRNA and control siRNA were purchased from Riobio Company (Guangzhou, China). The sequence was a listed in Supplementary Table S1.

**Luciferase reporter gene assays**

Luciferase reporter gene assays were performed using the Dual-Luciferase Reporter Assay System (Promega) according to the manufacturer’s instructions. Briefly, cells were cultured in 24-well plates at 3 × 10^4^ cells per well. After 24 hours, the cells were transiently co-transfected with the pRL-TK plasmid (Promega) containing the Renilla luciferase gene, which is used for internal normalization, and with various constructs containing the two 500-600 base pair fragments of ACBP promoter. All experiments were performed at least three times.

**RNA isolation, reverse transcription-polymerase chain reaction (RT-PCR), and quantitative reverse transcription Real-time PCR (qRT-PCR)**

Total RNA was extracted using Trizol reagent (Invitrogen, Carlsbad, CA). Complementary DNA was synthesized from total RNA using poly (A)-tailed total RNA and reverse transcription primer with ImPro-II Reverse Transcriptase (Promega, Madison, WI), according to the manufacturer's protocol. RT-PCR analysis was performed using specific primers for the ACBP gene. The RT-PCR was performed using DreamTaqTM Hot Start Green PCR Master Mix (Thermo Fisher Scientific, Carlsbad, CA) according to the manufacturer's protocol. The qRT-PCR was performed according to the instructions of Fast Start Universal SYBR Green Master (Rox) (Roche Diagnostics GmbH, Mannheim, Germany). All primers are listed in Supplementary Table 1. GAPDH was used as the controls, and TBP was used to normalize the results.

**Western blotting analysis**

The expression of ACBP was examined using Western blotting analysis. Ice-cold RIPA (Solarbio, Beijing, China) buffer supplemented with phosphatase and protease inhibitors was used for protein extraction. Total protein samples were separated via SDS-PAGE (12%) using the Bio-Rad Trans-Blot system and transferred to PVDF membranes. The primary antibodies used were mouse anti-GAPDH (Proteintech Group, Chicago, USA) at a dilution of 1:5000 and rabbit anti-ACBP (Proteintech Group, Chicago, USA) at a dilution of 1:500.

**Peripheral blood cell counts**

Peripheral blood obtained from the orbital sinus by using a micro-pipette coated with the anticoagulant K_2_EDTA. The cell counts, including white blood cell (WBC) counts, red blood cell (RBC) counts, hemoglobin (HGB), the percentage of neutrophil granulocytes (NE%) and the percentage of lymphocytes (LY%) were counted using a Celltac E hemocytometer (Nihon Kohden, Japan).

**Measure of spleens, thymus and colon**

C57BL/6J mice were treated with total body irradiation at a single dose of 4 Gy. After 21 days, the mice were euthanized, and the spleens and thymus were excised and measured. For colon measurement, C57BL/6J mice were treated with total abdominal irradiation at a single dose of 12 Gy. After 21 days, the mice were euthanized, and the colon tissues were excised and measured.

**Flow cytometry analysis**

Bone marrow cells were flushed from femurs with sterile phosphate-buffered saline (PBS). For HPC and HSC analysis, bone marrow cells were then filtered prior to staining with antibodies. Bone marrow cells were incubated with biotin-labeled antibodies specific for murine Ter119, B220, Gr1, CD11b, CD4, and CD8 (mixed Lineage antibodies) and then stained with streptavidin, c-kit, and Sca-1. Data acquisition was performed using a BD Accuri C6 and analyzed using BD Accuri C6 software (BD Bioscience, San Jose, CA, USA).

**FITC–dextran permeability experiments**

The mice were administrated with (or without) IPA following 12 Gy TAI. After 20 days, mice were fasted 14 h and treated with 60 mg per 100 g body weight of fluorescein isothiocyanate–dextran (FITC–dextran, FD4, 3000–5000 kD, Sigma-Aldrich, Madrid, Spain) in a volume of 0.2 ml. Blood samples were obtained by cardiac puncture at 4 h after administration of FITC-dextran and centrifuged at 860 g for 5 min at room temperature to obtain the serum. Fluorescence intensity of each serum sample (DTX 880 Multimode Detector, Beckman Coulter, CA, USA) was measured.

**Quantification of IL-6 and TNFɑ by ELISA**

Plasma was collected using K2EDTA as an anticoagulant. Centrifuge for 15 minutes at 1000g, 4 °C. Small intestine tissues were ground in saline. Sample dilutions of 1:4 were used for the determination of IL-6 and TNFɑ levels using ELISA kit (Mlbio, Shanghai, China) according to the manufacturer’s protocol. Optical density was read at 450nm (Rayto, Shenzhen, China).

**Measurement of Malondialdehyde**

The level of malondialdehyde (MDA) in the small intestine was determined using a detection kit from Solarbio (Solarbio, Beijing, China) according to the manufacturer's instructions. Levels of MDA were evaluated and calculated by the following formula, according to the manufacturer's instructions:

The levels of MDA (nmol) in the small intestine:

= 25.8 × (A532−A600)

**Plasmid injection techniques**

Various amounts of plasmid were diluted with the normal saline (0.9% NaCl) at room temperature. Mice were held immobile in a researcher's hand and then plasmid was rapidly injected into the retro-orbital sinus using a 5 mL injector needle.

**Bioluminescence imaging**

Bioluminescence imaging (BLI) was performed with an IVIS imaging system (IVIS Lumina II, PerkinElmer Waltham, MA, USA), which was composed of a highly sensitive cooled CCD camera mounted in a light-tight camera box. Images and measurements of bioluminescent signals were acquired and analyzed using Living Image software. Luciferase activity that was detected in vivo using the IVIS imaging system was digitized and electronically displayed as a pseudo color overlay onto a grayscale animal image.

**Supplementary table 1.**

List of primers used in this paper.

| **Gene** | **Primer** | **Sequence (5′-3′)** |
| --- | --- | --- |
| **Primers for PCR** | |  |
| *GAPDH* | forward | TGTTTCCTCGTCCCGTAGA |
|  | reverse | CAATCTCCACTTTGCCACTG |
| *TBP* | forward | ACCCTTCACCAATGACTCCTATG |
|  | reverse | TGACTGCAGCAAATCGCTTGG |
| *IL-6* | forward | TGTGCAATGGCAATTCTGAT |
|  | reverse | GGTACTCCAGAAGACCAGAGGA |
| *TNFɑ* | forward | TTCTCATTCCTGCTTGTGGCA |
|  | reverse | ACTTGGTGGTTTGCTACGACG |
| *Glut1* | forward | TATCCTGTTGCCCTTCTGC |
|  | reverse | CCGACCCTCTTCTTTCATCTC |
| *Pgk1* | forward | GGAAAACCTCCGCTTTCATGTA |
|  | reverse | GCCTTCTGTGGCAGATTCACA |
| *MDR1* | forward | TACGCCTACTATTACACCG |
|  | reverse | CATCAAACCAGCCTATCTC |
| *Homo-ACBP* | forward | GCTCGGATGAGGAGATGCTG |
|  | reverse | TTGGCCTTGCCCGTGAAGT |
| *Mus-ACBP* | forward | CTCAAGGGCAAAGCCAAGTG |
|  | reverse | GAGGAGGAGCAGAGGTTAAC |
| *Homo-PXR* | forward | TCTCCCACTTCCCACTCGTTC |
|  | reverse | AGGCCTCCTGGCTTCTCATC |
| *Mus-PXR* | forward | CTTTGTGGTTGGGAATAAAC |
|  | reverse | TTGTCCAGAACACGGTCAGGA |
|  |  |  |
| **Primer for sequencing** |  |  |
| 515F |  | GTGCCAGCMGCCGCGGTAA |
| 806R |  | GGACTACHVGGGTWTCTAAT |
| **Sequence for siRNA** |  |  |
| ACBP siRNA |  | TTCACGGGCAAGGCCAAGTGG |
|  |  |  |
| **Sequence for shRNA** |  |  |
| Sh-ACBP-1 |  | GGATCCCACCGCCGGGTCTATTTCCTCAGATTTC  AAGAGAATCTGAGGAAATAGACCCGGCAAGCTT |
| Sh-ACBP-2 |  | GGATCCCACCGGGTCTATTTCCTCAGATATCTTC  AAGAGAGATATCTGAGGAAATAGACCCAAGCTT |
| Sh-ACBP-3 |  | GGATCCCACCGGTCTATTTCCTCAGATATCCTTC  AAGAGAGGATATCTGAGGAAATAGACCAAGCTT |
| Sh-PXR-1 |  | GGATCCCACCGGAGGAAGATGGAGGTCTTCATT  CAAGAGATGAAGACCTCCATCTTCCTCCAAGCTT |
| Sh-PXR-2 |  | GGATCCCACCGCGTCATCAACTTCGCCAAAGTTC  AAGAGACTTTGGCGAAGTTGATGACGCAAGCTT |
| Sh-PXR-3 |  | GGATCCCACCGCTGCATAAGGAGGAGTATGTTTC  AAGAGAACATACTCCTCCTTATGCAGCAAGCTT |
|  |  |  |
| **Primers for clone** |  |  |
| PGL-ACBP-1 | forward | GGGGTACCGGAGATTAGGCTGTGTAAC |
|  | reverse | CCCTCGAGCGTTCTTTGACTTGCTTG |
| PGL-ACBP-2 | forward | GGGTACCAGGAAGAAGCCTCGGCTT |
|  | reverse | CCTCGAGACTGGCAAGCGCCTTTA |

**Supplementary figures and figure legends**

**
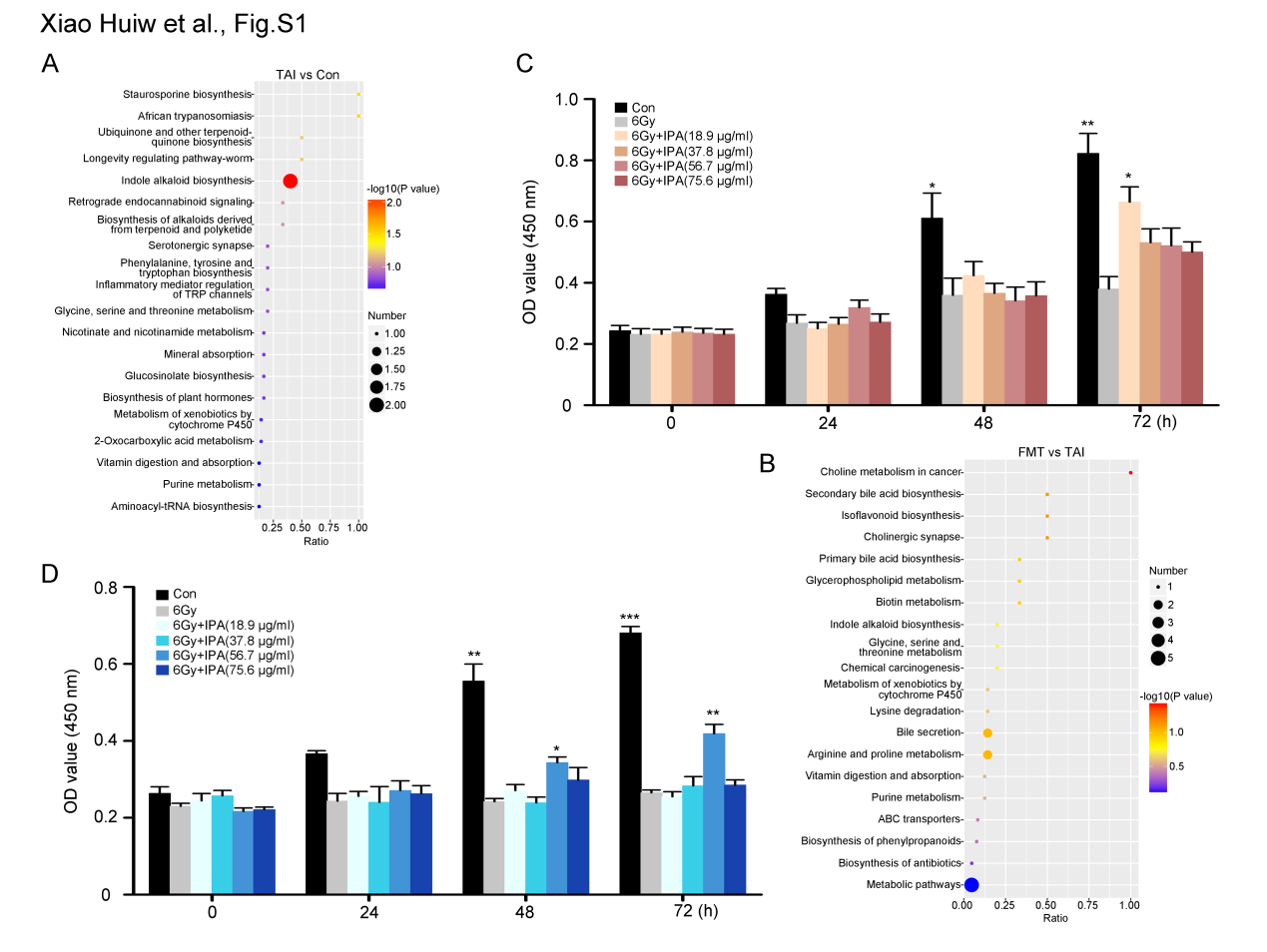
**

**Supplemental Figure 1.** IPA facilitated the proliferation of irradiated HIEC-6 cells and MODE-K cells. **A, B** Untargeted metabolomics KEGG analysis for control, 12 Gy TAI and TAI+FMT group. **C, D** The effects of concentration gradient IPA on the proliferation of MODE-K cells (C) and HIEC-6 cells (D) were assessed by CCK-8 assays. The data were presented as means ± SEM. Significant differences between each two cohorts are indicated: *P <0.05, **P < 0.01, ***P<0.005; Student’s *t*-test.


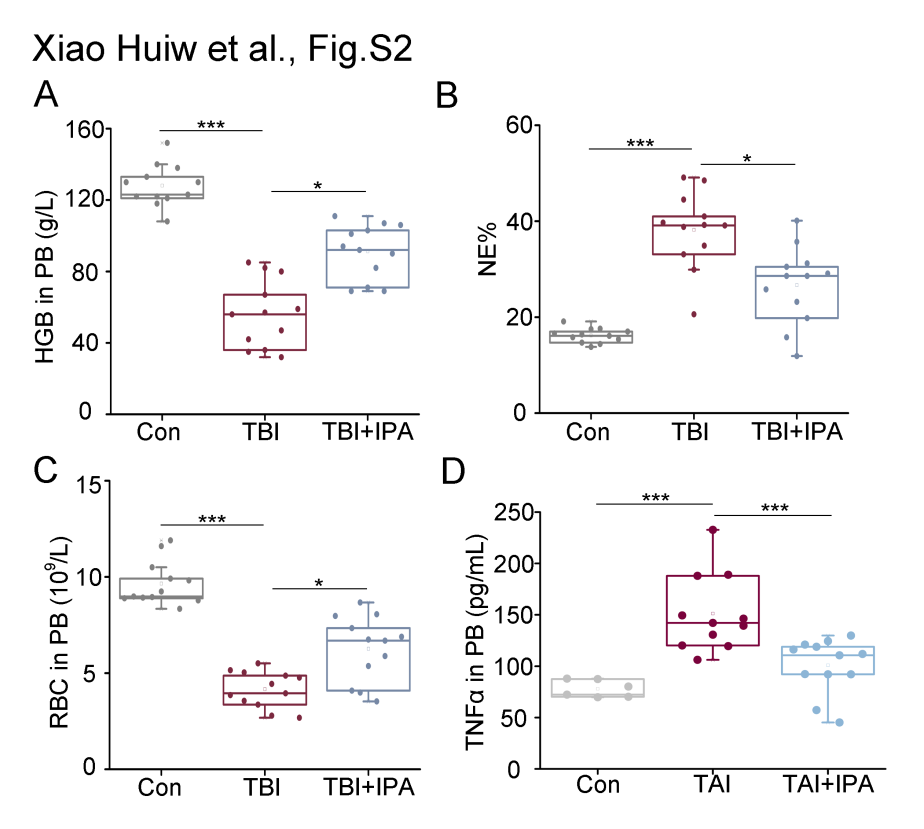


**Supplemental Figure 2.** Oral gavage of IPA ameliorates TBI-associated hematopoietic system injury. **A-C** Hemoglobin (HGB), percentage of neutrophil granulocytes (NE%) and red blood cell (RBC) in peripheral blood were measured at day 15 after 4 Gy TBI. The data were presented as means ± SEM (n = 12 per group). Significant differences between each two cohorts are indicated: *P < 0.05; ***P < 0.005; Student’s *t*-test. **D** The content of TNFɑ in PB was examined by ELISA. Mean ±SEM. Significant differences between each two cohorts are indicated: ***P <0.005; Student’s *t*-test, n=6 for control group; n=11 for TBI group; n=12 for TBI+IPA group.


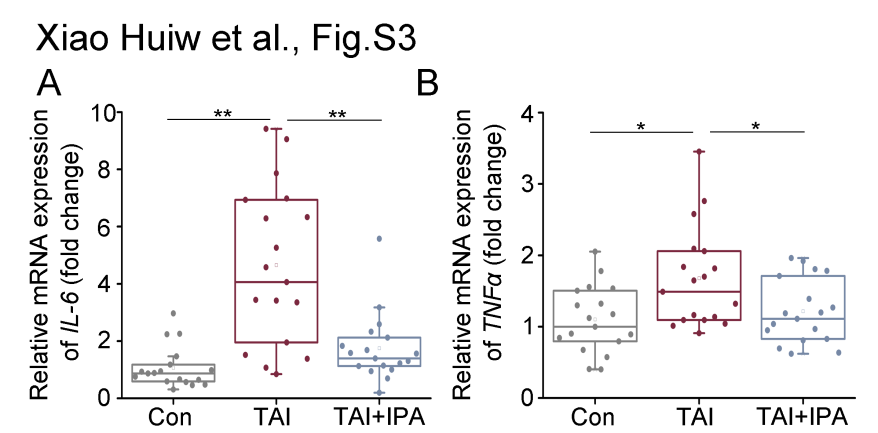


**Supplemental Figure 3.** IPA administration improves GI tract function after total abdominal irradiation. **A, B** The expression levels of *IL-6* and *TNFɑ* in small intestine tissues were examined by qRT-PCR. The small intestine tissues were obtained at day 21 after 12 Gy TAI. Mean ± SEM. Significant differences between each two cohorts are indicated: *P <0.05, **P < 0.01; Student’s *t*-test, n = 18 per group.


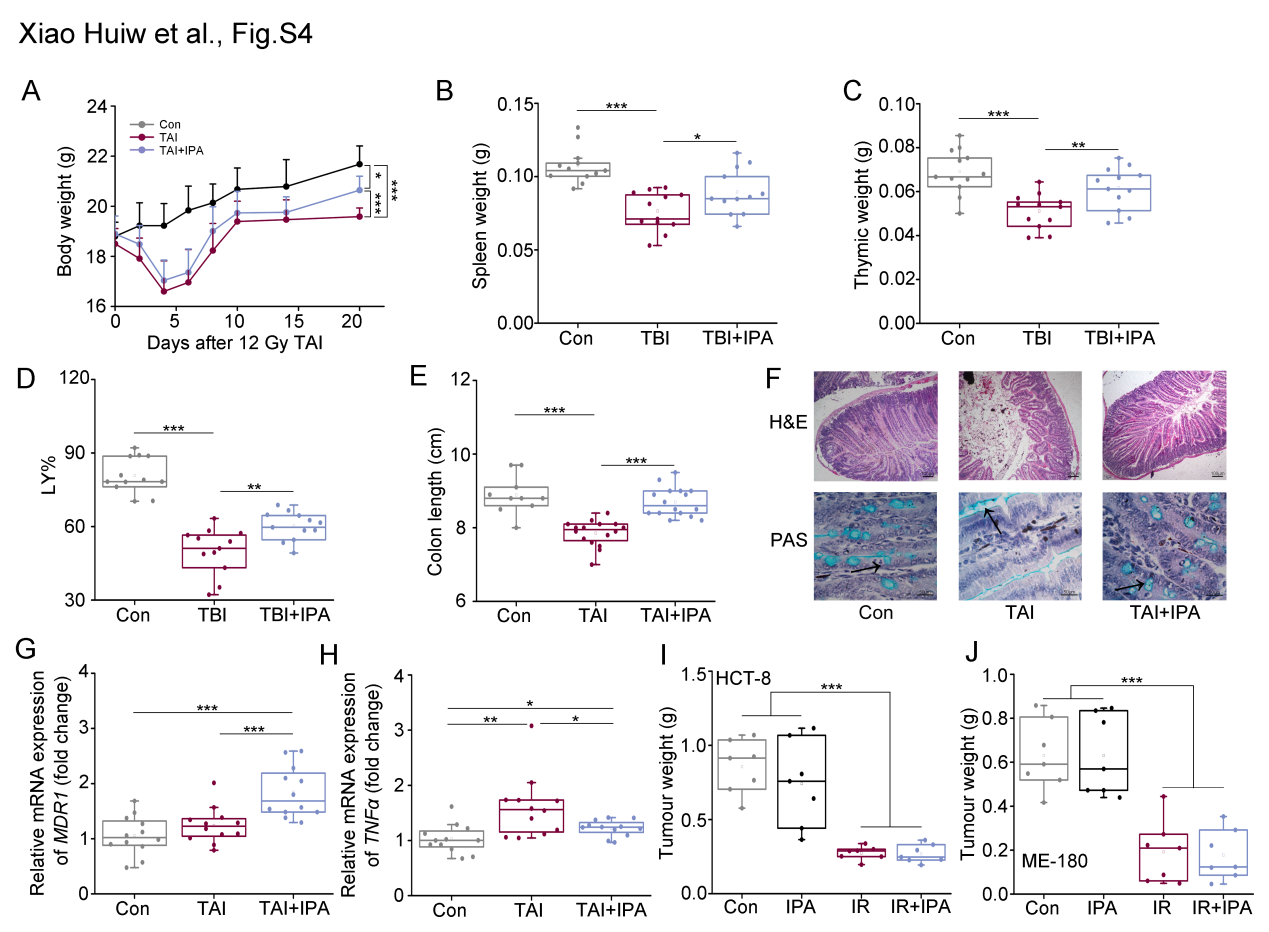


**Supplemental Figure 4.** IPA protects against radiation toxicity in mouse models without accelerating tumour growth. **A** Body weight was compared among three group mice after 12Gy TAI, n=24 per group; Significant differences between each two cohorts are indicated: *P <0.05, ***P<0.005; Student’s *t*-test. **B, C** The weight of dissected thymuses and spleens from female mice in the three groups, the thymuses and spleens were obtained at day 15 after 4 Gy TBI. Mean ±SEM. Significant differences between each two cohorts are indicated: *P <0.05, **P < 0.01, ***P < 0.005; Student’s *t*-test, n = 12 per group. **D** The percentage of lymphocytes (LY%) in PB were measured at 15 day after 4 Gy TBI. The data were presented as means ± SEM (n = 12 per group). Significant differences between each two cohorts are indicated: **P < 0.01; ***P < 0.005; Student’s *t*-test. **E** The length of dissected colon from female mice in the three groups, the colon tissues were obtained at day 21 after 12 Gy TAI. Mean ± SEM. Significant differences between each two cohorts are indicated: ***P < 0.005; Student’s *t*-test, n = 12 per group. **F** The morphology of the small intestine from female mice was shown by H&E (magnification: 100×; Scale bar: 100μm) and PAS (magnification: 1000×; Scale bar: 50μm) staining. The small intestine tissues were obtained at day 21 after 12 Gy TAI. The arrows point to the goblet cells. **G, H** The expression levels of *MDR1* (G) and *TNFɑ* (H) were examined in small intestine tissues by qRT-PCR. The small intestine tissues were obtained at day 21 after 12 Gy TAI. Mean ± SEM. Significant differences between each two cohorts are indicated: *P <0.05, **P < 0.01, ***P < 0.005; Student’s *t*-test, n = 12 per group. **I, J** Tumour weight from mice measured at the end of experiment. Data are expressed as mean ± SEM from 7 mice. Significant differences between each two cohorts are indicated: ***P < 0.005; Student's *t*-test.

**
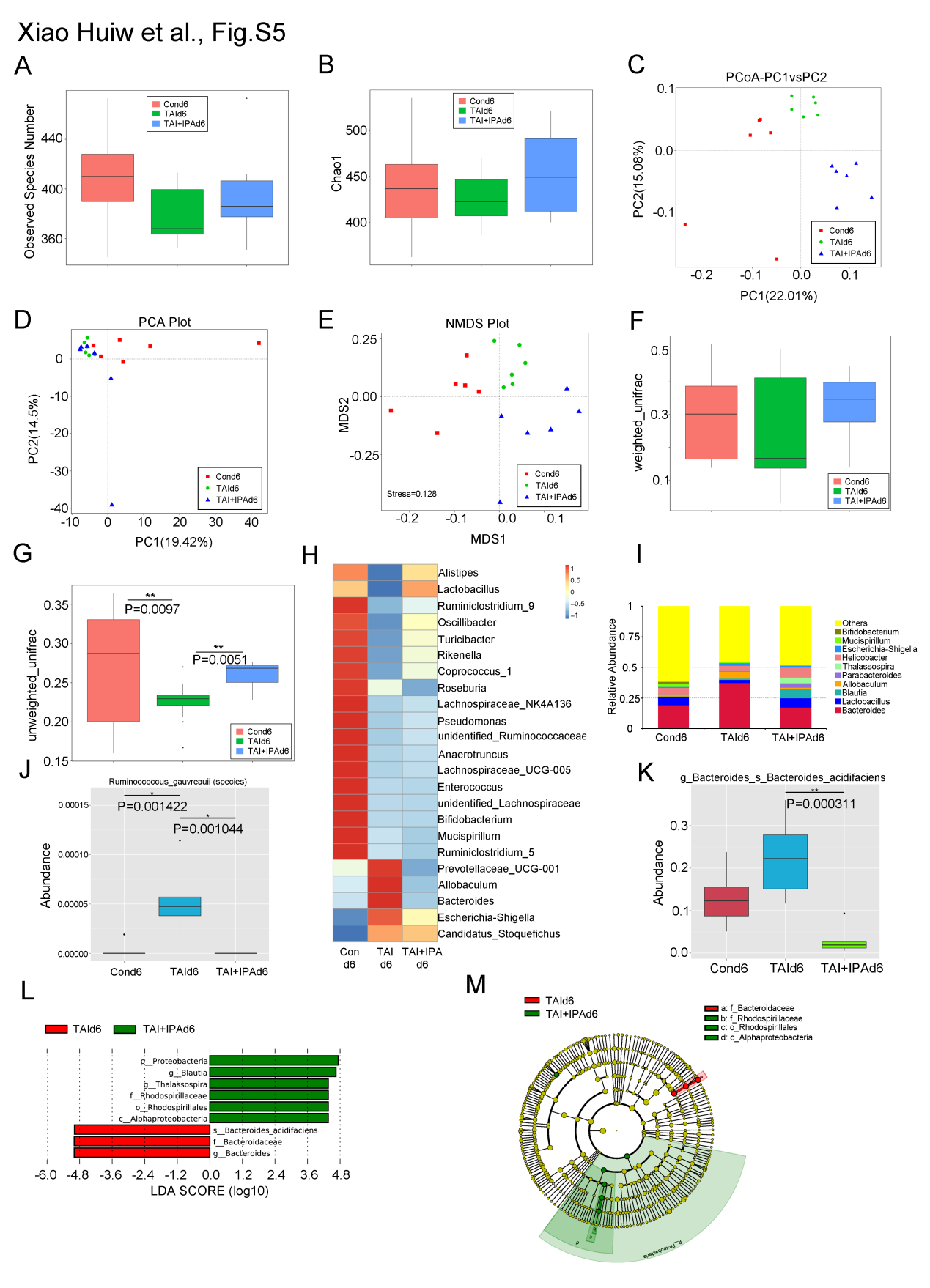
**

**Supplemental Figure 5.** IPA treatment changes irradiation-shaped intestinal bacterial structure at day 6 after TAI. **A, B** The observed species number and Chao1 diversity index of intestinal bacteria was examined by 16S rRNA high-throughput sequencing after 6 days of TAI exposure. Significant differences are indicated: Wilcoxon rank sum test. The top and bottom boundaries of each box indicate the 75^th^ and 25^th^ quartile values, respectively, and lines within each box represent the 50^th^ quartile (median) values. Ends of whiskers mark the lowest and highest diversity values in each instance. n = 6 per group. **C-E** PCoA, PCA and NMDS were used to measure the shift in intestinal bacterial composition profile after irradiation at day 6. **F, G** The β diversity of intestinal bacteria was compared by the weighted and unweighted Unifrac analysis. Significant differences are indicated: Wilcoxon rank sum test. n = 6 per group. **H** The alteration of intestinal bacterial patterns at the genus level was assessed by 16S rRNA sequencing, n=6 per group. The heat map is color-based on row Z-scores. The mice with the highest and lowest bacterial level are in red and blue, respectively. **I** The relative abundance of the top 10 bacteria at the genus level in Con and TAI-treated mice (with or without IPA oral gavage) was assessed using 16S high-throughput sequencing after irradiation at day 6, n=6. **J, K** The abundance of most varied strain bacteria was assessed using 16S high-throughput sequencing after irradiation at day 6. Significant differences are indicated: Wilcoxon rank sum test. n = 6 per group. **L** Linear discriminant analysis (LDA) effect size (LEfSe) results represented significantly different in abundance of gut bacteria between the TAI and IPA groups and indicated the effect size of each differentially abundant bacterial taxon in the small intestine after irradiation at day 6, n=6 per group. Significant differences are indicated: Wilcoxon rank sum test. **M** Linear discriminant analysis (LDA) effect size (LEfSe) tree results showed the significantly different of bacteria in abundance between TAI and IPA groups and indicated the effect size of each differentially abundant bacterial taxon in the small intestine after irradiation at day 6, n=6.


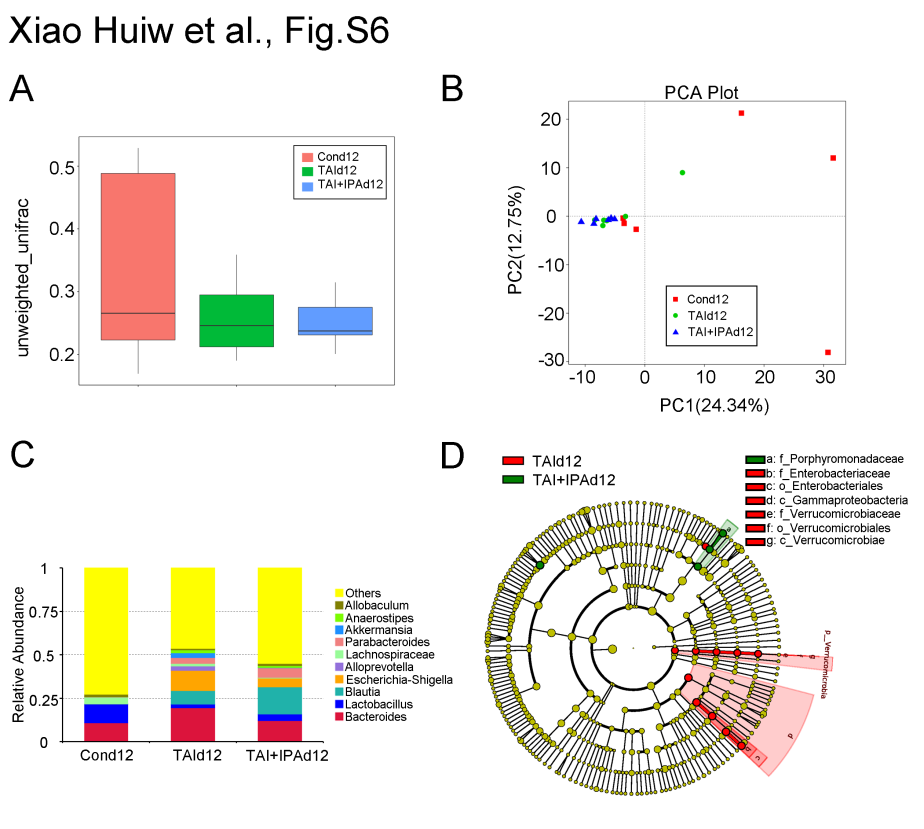


**Supplemental Figure 6.** IPA preserves irradiation-shifted enteric bacterial composition at day 12 after TAI. **A** The β diversity of intestinal bacteria was compared by the unweighted Unifrac analysis. Statistically significant differences are indicated: Wilcoxon rank sum test. n = 6. **B** Principal component analysis (PCA) was used to measure the shift in intestinal bacterial composition profile after irradiation at day 12, n=6 per group. **C** The relative abundance of the top 10 bacteria at the genus level in Con and TAI-treated mice (with or without IPA oral gavage) was assessed using 16S high-throughput sequencing after irradiation at day 12, n=6. **D** Linear discriminant analysis (LDA) effect size (LEfSe) tree results showed that the bacteria were significantly different in abundance between the TAI and IPA groups and indicated the effect size of each differentially abundant bacterial taxon in the small intestine after irradiation at day 12, n=6.


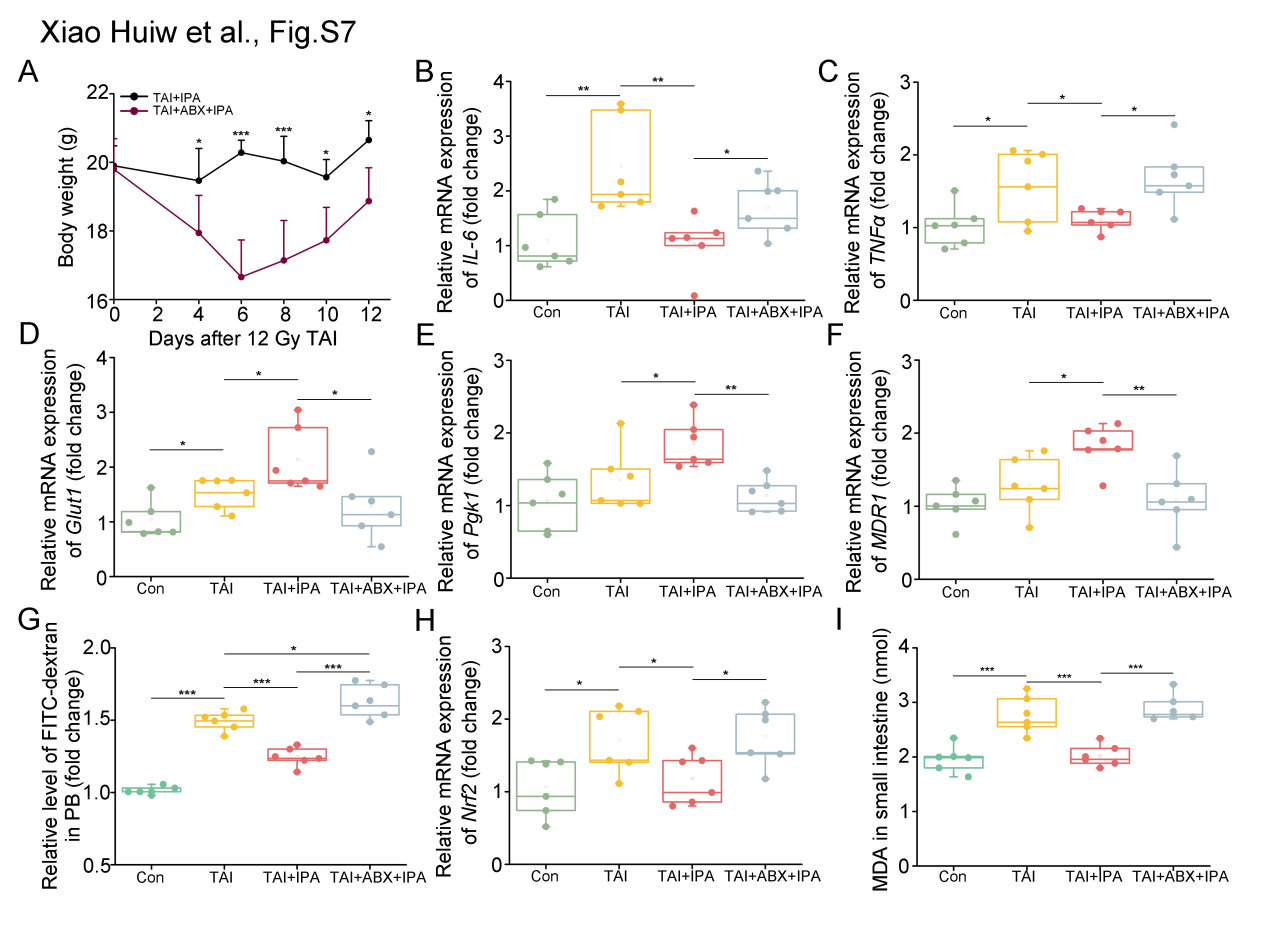


**Supplemental Figure 7.** Impact of antibiotics (ABX) and IPA on GI tract function after total abdominal irradiation. **A** Body weights were compared between the two group mice after 12 Gy TAI, n=24 per group; *P <0.05, ***P<0.005; Student’s *t*-test. **B-F** The expression levels of *IL-6* (B), *TNFɑ* (C) *Glut1* (D), *Pgk1* (E) and *MDR1* (F) in small intestine tissues were examined by qRT-PCR. The small intestine tissues were obtained at day 21 after 12 Gy TAI. Mean ± SEM. Significant differences between each two cohorts are indicated: *P <0.05, **P < 0.01; Student’s *t*-test, n = 6 per group. **G** The FITC-dextran in PB was assessed at day 21 after 12 Gy TAI. Mean ± SEM. Significant differences between each two cohorts are indicated: *P < 0.05, ***P < 0.005; Student’s *t*-test, n = 6 per group. **H** The expression level of *Nrf2* was assessed in small intestine tissue by qRT-PCR. The small intestine tissues were obtained at day 21 after 12 Gy TAI. Mean ± SEM. Significant differences between each two cohorts are indicated: *P <0.05; Student’s *t*-test, n = 6 per group. **I** The content of MDA in small intestine tissues were examined. Mean ± SEM. Significant differences between each two cohorts are indicated: ***P<0.005; Student’s *t*-test, n=6 per group.

**
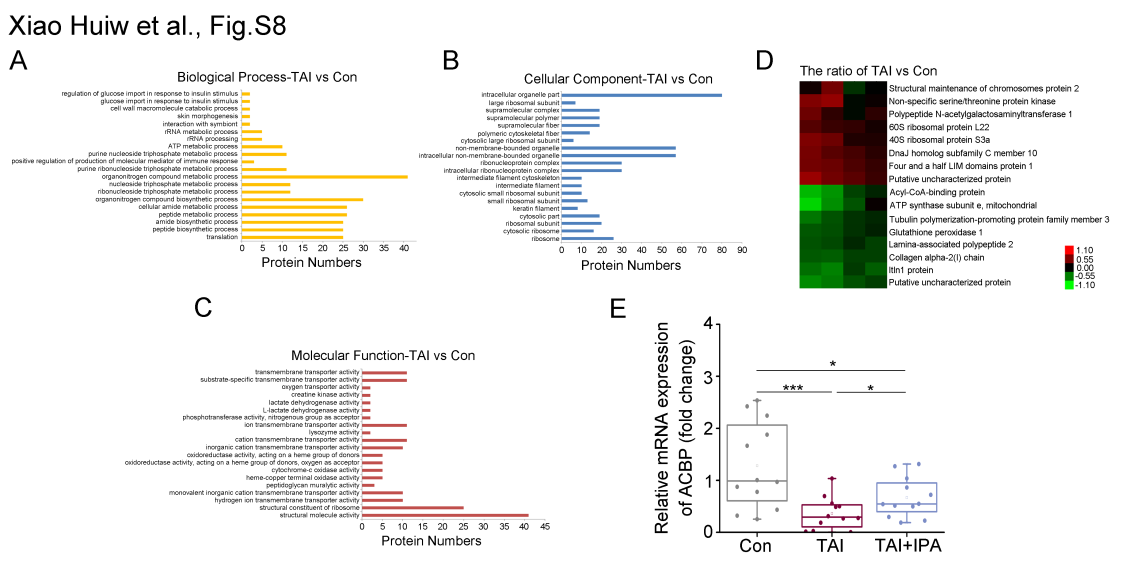
**

**Supplemental Figure 8.** TAI changes the protein expression profile of small intestine. **A-C** Bioinformatics analysis of different proteins in small intestine of irradiated mice compared to the control group through gene ontology (GO) in biological process (A), cellular component (B) and molecular function (C). Information on the number of involved proteins in a term is shown on the x-axes. **D** Hierarchical cluster analysis for the different proteins in small intestine of irradiated mice compared to the control group. **E** The expression level of ACBP was examined in small intestine tissues of female mice by qRT-PCR. Mean ± SEM. Significant differences between each two cohorts are indicated: *P <0.05, ***P<0.005; Student’s *t*-test, n = 18 per group.


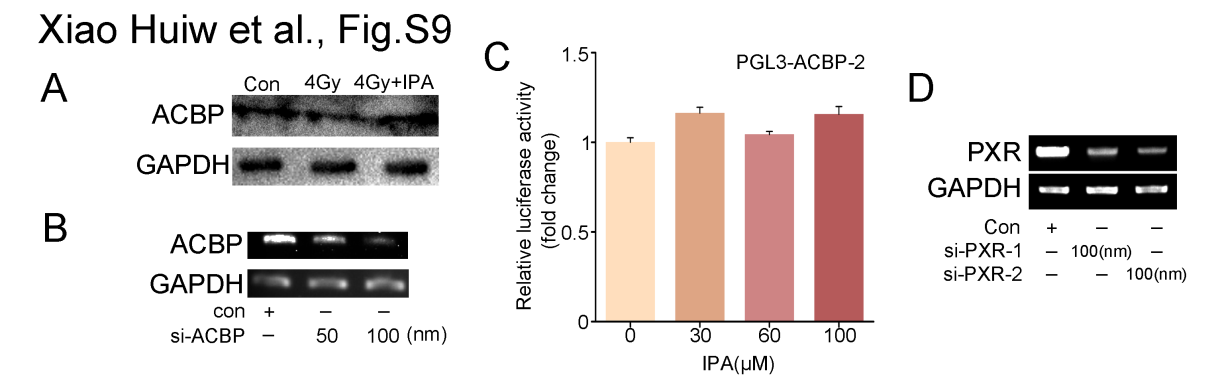


**Supplemental Figure 9.** ACBP contributes to the protective function of IPA toward irradiation *via* PXR. **A** The expression of *ACBP* was examined by western blotting in HIEC-6 cells, irradiated HIEC-6 cells and irradiated HIEC-6 cells cultured with IPA (37.8 µg/mL). **B** The interference efficiency of si-ACBP toward ACBP mRNA was assessed by RT-PCR. **C** The effect of IPA on PGL3-ACBP-2 reporter was measured by luciferase reporter gene assays in HIEC-6 cells, Significant differences between each two cohorts are indicated: Student’s *t*-test. **D** The interference efficiency of si-PXR toward PXR mRNA was assessed by RT-PCR.


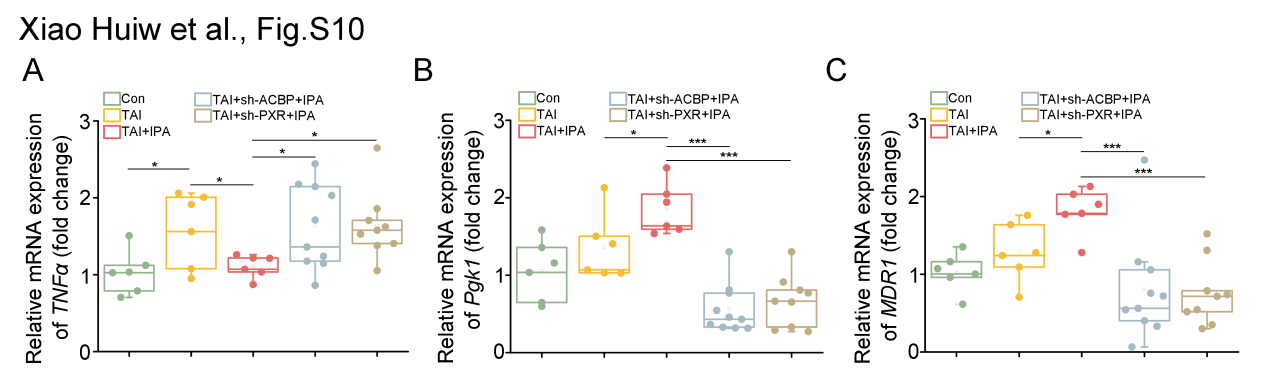


**Supplemental Figure 10.** ACBP or PXR inhibition blocked the protective function of IPA toward irradiation. **A-C** The expression level of *TNFɑ*, *Pgk1* and *MDR1* was examined in small intestine tissues by qRT-PCR. The small intestine tissues were obtained at day 21 after 12 Gy TAI. Significant differences between each two cohorts are indicated: *P <0.05, ***P <0.005; Student’s *t*-test, n = 6 for control group, n=6 for TAI group, n=6 for TAI+IPA group, n=10 for TAI+sh-ACBP+IPA group, n=9 for TAI+sh-PXR+IPA group.


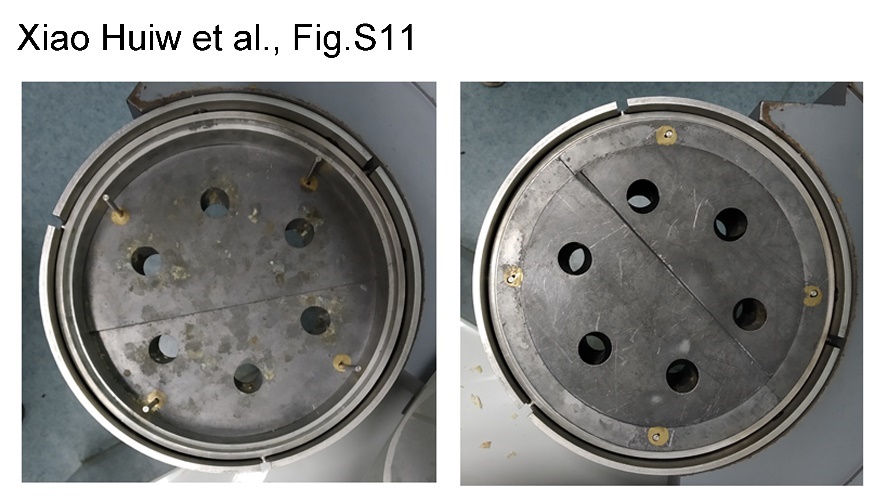


**Supplemental Figure 11.** Photographs of the lead shielding apparatus used in this study. The left photograph showed the lead shielding without lid, and the right photograph showed the lead shielding with lid.
